# Dynamic Shifts in the HIV Proviral Landscape During Long Term Combination Antiretroviral Therapy: Implications for Persistence and Control of HIV Infections

**DOI:** 10.3390/v12020136

**Published:** 2020-01-25

**Authors:** Elizabeth M. Anderson, Francesco R. Simonetti, Robert J. Gorelick, Shawn Hill, Monica A. Gouzoulis, Jennifer Bell, Catherine Rehm, Liliana Pérez, Eli Boritz, Xiaolin Wu, Daria Wells, Stephen H. Hughes, Venigalla Rao, John M. Coffin, Mary F. Kearney, Frank Maldarelli

**Affiliations:** 1 HIV Dynamics and Replication Program, NCI, NIH, Frederick, MD 21702, USA; Elizabeth.Anderson@pennmedicine.upenn.edu (E.M.A.); s.aeruginosa@gmail.com (F.R.S.); hillshaw@mail.nih.gov (S.H.); monica.gouzoulis@nih.gov (M.A.G.); hughesst@mail.nih.gov (S.H.H.); kearneym@mail.nih.gov (M.F.K.); 2 Department of Biology, The Catholic University of America, Washington, DC 20064, USA; rao@cua.edu; 3 Frederick National Laboratory for Cancer Research, Frederick, MD 21702, USA; gorelicr@mail.nih.gov (R.J.G.); jennifer.bell@nih.gov (J.B.); 4 Laboratory of Immunoregulation, NIAID, NIH, Bethesda, MD 20814, USA; crehm@niaid.nih.gov; 5 Virus Persistence and Dynamics Section, VRC, NIAID, NIH, Bethesda, MD 20814, USA; liliana.perezrodriguez@nih.gov (L.P.); boritze@niaid.nih.gov (E.B.); 6 Cancer Research Technology Program, Leidos Biomedical Research, Inc., Frederick National Laboratory for Cancer Research, Frederick, MD 21702, USA; forestwu@mail.nih.gov (X.W.); wellsd2@mail.nih.gov (D.W.); 7 Department of Biology, Tufts University, Boston, MA 02155, USA; john.coffin@nih.gov

**Keywords:** HIV clonal expansion, proviruses, ddPCR, HIV persistence

## Abstract

Combination antiretroviral therapy (cART) controls but does not eradicate HIV infection; HIV persistence is the principal obstacle to curing infections. The proportion of defective proviruses increases during cART, but the dynamics of this process are not well understood, and a quantitative analysis of how the proviral landscape is reshaped after cART is initiated is critical to understanding how HIV persists. Here, we studied longitudinal samples from HIV infected individuals undergoing long term cART using multiplexed Droplet Digital PCR (ddPCR) approaches to quantify the proportion of deleted proviruses in lymphocytes. In most individuals undergoing cART, HIV proviruses that contain *gag* are lost more quickly than those that lack *gag*. Increases in the fraction of *gag*-deleted proviruses occurred only after 1–2 years of therapy, suggesting that the immune system, and/or toxicity of viral re-activation helps to gradually shape the proviral landscape. After 10–15 years on therapy, there were as many as 3.5–5 times more proviruses in which *gag* was deleted or highly defective than those containing intact *gag*. We developed a provirus-specific ddPCR approach to quantify individual clones. Investigation of a clone of cells containing a deleted HIV provirus integrated in the *HORMAD2* gene revealed that the cells underwent a massive expansion shortly after cART was initiated until the clone, which was primarily in effector memory cells, dominated the population of proviruses for over 6 years. The expansion of this HIV-infected clone had substantial effects on the overall proviral population.

## 1. Introduction

Successful combination antiretroviral therapy (cART) completely blocks ongoing viral replication [1,2,3] and improves infection-associated morbidity and mortality, but does not cure the infection [4,5,6,7]. Low-level plasma viremia persists in most patients on cART even after years of treatment [8,9,10,11]. The source of the virus is uncertain, but is likely to be long-lived cells that carry infectious HIV proviruses [12,13]. Although the majority of the infected cells in patients on long-term cART carry defective proviruses (>98%), a small subset contains intact replication-competent proviruses that can give rise to rebound virus if cART is interrupted [14,15]. HIV persistence despite therapy is the major barrier to a cure. It is likely that persistence is due to the long-term survival and growth of HIV-infected cells that were present prior to the initiation of cART [1,2,3,16,17]. 

The initiation of cART blocks new rounds of virus infection, resulting in substantial shifts in the populations of infected cells. When cART is initiated, HIV viremia declines with multiphase kinetics, reflecting the loss of different populations of infected cells that were producing the plasma virus. The majority of HIV in plasma (>99%) is produced by short-lived CD4+ lymphocytes (half-life: around 1 day) [18,19,20]. Subsequently, the remaining viremia decays more slowly, in at least three successive phases with half-lives of 14 days, 39 weeks, and 110 weeks, respectively [11,19,21]. Most patients have relatively stable residual low-level viremia after the last phase of decay [22]. On average, plasma HIV levels decline >14,000-fold within 1–2 years after cART is initiated [22]. In contrast to the profound decrease in viremia, the decline in the number of HIV-infected cells is much more modest. The number of HIV-infected cells declines approximately 10- to 30-fold during the first several years of cART [23]. Studies by Siliciano and colleagues, and Crooks and colleagues, have reported that the population of infected CD4+ T cells that can give rise to infectious virus following activation in vitro also decays slowly, with an average half-life of 43–44 months [13,24]. 

The majority of proviruses in individuals on cART are noninfectious due to defects including deletions, mutations, and/or extensive G-to-A mutations caused by APOBEC-mediated cytidine deamination [14,15,25]. HIV-infected cells with defective proviruses cannot produce infectious virus, but these cells remain central to understanding what happens in patients, because defective proviruses can produce viral RNA and protein and are potential sources of immune activation [26,27,28,29]. Previously, we and others reported that infected cells, including cells that carry infectious proviruses, can undergo clonal expansion [30,31,32], and can divide without viral reactivation in vitro and in vivo [33,34]. During cART, at least 40% of the infected cells are the result of clonal expansion [16,31,32]. However, only a minor portion of the cells in infected cell clones contain HIV RNA; the majority of the cells carry latent proviruses [35]. The loss of infected cells that express viral RNA and proteins, coupled with the clonal expansion of a small fraction of infected cells, is likely to have a substantial effect on the structure of HIV provirus populations during the course of infection [36]. 

In the setting of long-term therapy, populations of HIV-infected cells undergo substantial shifts. HIV-infected cells can clonally expand, and the clones can subsequently decrease in size [16,37,38]. Siliciano and coworkers reported that the fraction of defective proviruses increases during cART [14,15], and Pinzone et al. observed that, on average, the fraction of the proviruses that are intact declines slowly during cART [28], despite a relatively constant total proviral load. These shifts in HIV populations take place in the setting of ongoing immune recovery and increases in CD4+ T cells after cART is initiated. The increases in CD4+ T cells in blood during the first several months of cART have been largely attributed to a redistribution of memory CD4+ T cells from tissue compartments [39,40]. Subsequent increases in CD4+ T cells in the blood during cART include increases in the number of naïve cells [41,42,43], and are likely to be a result of partial immune reconstitution. Immune activation persists throughout cART and is characterized by elevated levels of soluble markers, such as IL-6, which are linked to long-term morbidity and mortality during cART [44]. HIV-infected cells can be eliminated or undergo clonal expansion during this process, but the dynamics of the proliferation and survival of infected cells during prolonged antiretroviral therapy are not well understood.

Longitudinal analyses of HIV proviral dynamics will yield critical information on the timing of the shifts in the populations of HIV-infected cells that take place during cART. There are published reports that measure viral DNA load by estimating the numbers of intact proviruses [3,28] and measuring levels of proviruses that are likely to be infectious [45]; in addition, there are methods that quantify either internal portions of the HIV-1 genome or the HIV-1 long terminal repeats (LTRs), but not both simultaneously [23,46,47,48,49,50,51]. We have developed Droplet Digital PCR (ddPCR) approaches that make it possible to simultaneously quantify both HIV-1 LTRs and internal HIV-1 DNA sequences; these methods are sensitive enough to measure small (2- to 3-fold) differences in relative abundance [52]. We also developed specific primer:probe sets to measure the levels of individual abundant infected clones using primers that flank the site of integration and the provirus. Here we quantify the dynamics of HIV proviral populations, both total and deleted, prior to and following the initiation of cART, using longitudinal samples from a cohort of individuals who initiated cART and were followed for c. 9 years. To characterize HIV populations during CD4+ T cell recovery, we quantified HIV-infected cell populations in two ways: By determining the total concentration of proviruses in blood cells (HIV DNA/mL blood) and by determining the fraction of HIV-infected cells in the CD4+ T cell population (HIV DNA/10^6^ CD4+ T cells). We found that populations of HIV-infected cells shift during cART with a progressive enrichment in the fraction of deleted proviruses that becomes detectable only after 1–2 years on therapy. These findings shed new light on the dynamics of the population of HIV-infected cells during cART and have implications for the understanding of persistence of viral DNA and ongoing pathogenesis. These findings also have relevance to efforts to control or cure HIV, because the populations of infected cells targeted by eradication strategies will be fundamentally different depending on timing of these interventions.

## 2. Materials and Methods

### 2.1. Study Approval

Patient-derived material was collected from HIV-infected individuals who were enrolled in a natural history study of the effects of antiretroviral therapy in HIV-infected adults (clinical protocol 97-I-0082) at the National Institutes of Health (NIH) Clinical Center in Bethesda MD [1,53,54] (Table S1). Participants provided written informed consent under protocols approved by the NIAID Institutional Review Board (FWA00005897). Participants were over the age of 18 at study entry and reported no prior cART. Therapy was initiated during 1997–2000 with a four-drug combination regimen consisting of 2 NRTIs + nevirapine + indinavir, and patients underwent frequent phlebotomy to investigate HIV-1 viral decay kinetics [54]. 

### 2.2. Samples

Patient-derived blood samples were collected prior to and after the initiation of cART. Total peripheral blood mononuclear cells (PBMC) obtained by phlebotomy or leukapheresis were separated on a Ficoll gradient and stored frozen. The concentration of CD4+ T cells (cells/mm^3^) was measured by flow cytometry. Plasma was prepared from phlebotomy samples, and HIV-1 RNA concentrations were quantified by bDNA [55] or PCR (Amplicor, Roche Diagnostic Systems, Branchburg, NJ, USA) with a lower limit of detection of 50 copies/mL plasma. 

### 2.3. CD4+ T Cell Subset Separation

PBMC were stained with viability dye and the following fluorescently-labeled antibodies: CD3-APC-H7, CD4-BV785, CD8-QD655, CD11c-PE, CD14-PE, CD27-PC5, CD45RO-ECD, CD56-APC, CD57-BV421, CCR7-Alx700, and TCRg/d-APC. CD4+ T cell subsets were then sorted on a FACSAria (Becton Dickinson, Franklin Lakes, NJ, USA) using previously described protocols [56]. Viable cells that were CD3+CD8-CD4hi and negative for lineage-exclusion markers CD56, TCRgd, CD14, and CD11c were sorted into three populations: CD27+CD45RO-CCR7+CD57- cells, which include naïve and stem cell memory (N/SCM) subsets; CD27+CD45RO+ cells, which include central and transitional memory (C/TM) subsets; and CD27- cells, which represent the effector memory (EM) subset [56]. The sorting strategy is detailed in Supplemental Figure S7. Cells sorted were then pelleted and kept at −80 °C until DNA isolation.

### 2.4. DNA Isolation

Total DNA was extracted from approximately two million PBMC using an in-house extraction protocol, as previously described [57]. In brief, cryopreserved PBMC were thawed and diluted with 1mL warmed RPMI medium added dropwise (GIBCO, Gaithersburg, MD, USA). Two million cells were diluted further with warmed RPMI and centrifuged at 500× *g* for 5 min. Cell pellets were snap-frozen and stored at −80 °C prior to genomic DNA extraction. Two million cells were lightly thawed at room temperature and 100 μL of 3 M guanidinium hydrochloride (Sigma, St. Louis, MO, USA) containing 50 mM Tris-HCl pH 7.6, 1 mM calcium chloride and 100 μg proteinase K (Ambion, Austin, TX, USA) was added to the cell pellet. To ensure complete cell lysis, the mixture was pulse vortexed before a 1 h incubation in a 42 °C water bath. Following the incubation, 400 μL of 6 M guanidinium thyiocynate (GuSCN) containing 50 mM Tris HCl pH 7.6, 1 mM EDTA, and 600 μg/mL glycogen (Sigma, St. Louis, MO, USA) was added. Samples were incubated again at 42 °C for 10 min. Total nucleic acids were precipitated by adding 500 μL of 100% isopropanol, vortexing at high intensity for 10 s, and spinning at 21,000× *g* for 10 min. The supernatant was removed and pellets were stored in 750 μL 70% ethanol for downstream applications.

### 2.5. Total HIV-1 DNA Quantification

DNA extracted from PBMC was quantified using ddPCR assays targeting HIV-1 *gag,* LTR, and *tat/rev* regions, and a host gene (*CCR5*) as previously described [52] (Table S4). To this end, total genomic DNA pellets stored in ethanol were spun at 21,000× *g* for 10 min and supernatant was removed. The sample was air dried to remove residual ethanol until pellets were just translucent. Nucleic acid (NA) pellets were resuspended in 150 μL Tris-HCl pH 8.0 and sonicated with a Branson ultrasonic cup horn sonifier (Emerson, St. Louis, MO, USA) at 60% amplitude in pulse mode for 5 s, then pulse vortexed, spun, and repeated three times. Resuspended NA were then heated in a Thermomixer (Eppendorf, Hamburg, Germany) at 100 °C for 15 min, then snap cooled on ice, and stored at −20 °C until assayed. DNA was assayed in triplicate on the Droplet Digital PCR platform (Bio-Rad, Hercules, CA, USA) for various sequences. A 20 μL PCR master mix was made to a final concentration of 1 × ddPCR Supermix for probes (Bio-Rad), 750 nM forward and reverse primers, 250 nM probe, 5 μL of DNA template, and molecular grade water.

Total cell DNA was measured by ddPCR as *CCR5* DNA copies/well with previously reported primers and probes [58]. Total HIV-1 DNA (detecting both integrated and unintegrated HIV-1 DNA) was measured using a forward primer in R of the HIV LTR (RU5-F), a reverse primer in U5 (RU5-R), and a HEX tagged probe in U5 modified with an internal ZEN quencher (RU5-Probe). This assay was multiplexed with previously published oligos in p24 of *gag* [59] modifying the *gag* 32t probe to contain an internal ZEN quencher and a 3′ Iowa Black FQ (HIV SCA probe 32t ZEN). The first exon of *tat* was quantified using the forward primer TatRev1_F, reverse primer TatRev1_R, and a Hex tagged TatRev1_probe [52]. The second exon of *tat* and *rev* was measured with a forward primer in HIV *env* called TatRev2_F, a reverse primer in exon 2 of *tat/rev:* msRNA-R, and a FAM tagged probe in exon 2 of *tat/rev*: msRNA Probe [52]. All ddPCR primer sequences are listed in Table S4, oligos were ordered from Integrated DNA Technologies.

To optimize the ddPCR assay we analyzed ACH2 cells, a chronically-infected cell line containing approximately 1 HIV provirus per cell, and CEM cells, an HIV-uninfected T lymphoblastoid cell line. We performed 26 individual ddPCR runs (N = 64 replicates) of approximately 27,000 cells per well. The number of proviruses per cell can be determined by dividing the number of LTR copies by the number of *CCR5* copies; each cell has 2 copies of *CCR5*; each provirus has 2 LTRs. We found an average of 1.2 proviruses per ACH2 cell, a value that is significantly higher than the reported single integrated provirus (one sample t-test *p* = 0.0003). These data are consistent with recent findings that there is ongoing HIV replication with re-infection of ACH2 cells in vitro [60,61]. The average ACH2 LTR:*gag* ratio was 1.8 (standard deviation: 0.19, range: 1.4–2.3), this ratio is slightly but significantly less than the expected 2:1 ratio (one sample t-test *p* < 0.0001). The reason for the < 2:1 ratio is uncertain but may be due to the contribution of 1 and 2 LTR circles to HIV quantification. ACH2 cells contain readily detectable 2 LTR circles [62] and it is possible that the process of shearing yields fragments with both LTRs that are subsequently incorporated into a single droplet, and quantified as a single LTR. To investigate whether false positive droplets were contributing to the ddPCR results, which can complicate low level detection [63,64,65,66], we investigated the uninfected CEM cell line. There were few false positive droplets in DNA from uninfected CEM cells (<1 copy in 60,000 CEM cells), demonstrating that false positive droplets were not contributing to the ddPCR signal.

Provirus specific ddPCR assays were developed to selectively quantify specific proviruses and assess the abundance of expanded clones in two individuals. These assays combined the LTR forward primer in R and the LTR probe in U5 described above (RU5-F and RU5-Probe) with provirus-specific primer designed on an individual basis. We focused on two abundant proviruses, one present in the *HORMAD2* gene, which accounts for approximately 20% of all proviruses in one patient, and an infectious provirus, AMBI-1, which accounted for 3.1% of proviruses in another [16,30]. The *HORMAD2* provirus specific ddPCR assay was designed utilizing an overlapping forward primer that crosses the host-HIV junction at the *HORMAD*2 integration site previously described in participant AVBIO2_21 [16] (HORMAD2_FOOL_01). An *AMBI-1* provirus-specific ddPCR assay was developed to measure a previously described cell clone harboring a replication-competent provirus [16,30] using an overlapping forward primer that crosses the U5 of the 3′ LTR into the ambiguous integration site (AMB1_ROOL_01). The sequence of the host portion of this primer was obtained from the sequence of the integration site initially identified (16). As previously described, the position of this host sequence in the human genome cannot be unequivocally identified because of the presence of a number of similar sequences (pseudogenes and the like); hence the clone was denoted “AMBI” for “ambiguous”. This clone, though abundant, was not detected with this ddPCR strategy. These data demonstrate that substantial clonal abundance (>3.1%) is necessary to quantify individual proviruses.

To quantify total proviruses in an individual we first determined the total number of proviruses in blood volume using the Nadler formula for blood volume [67]. Because blood contains <2% of all CD4+ T cells [68], we calculated total numbers of proviruses in the whole body assuming that 2% of infected cells are in blood.

End-point ddPCR was performed with the following conditions: 95 °C for 10 min, then 40 cycles of 94 °C for 30 s, 55 °C for 1 min with a ramp rate of 2 °C/second, followed by a final 98 °C for 10 min and a held at 12 °C. Assay products were then read on the QX200 Droplet Reader (Bio-Rad) and were analyzed using the Quantasoft software version 1.7.4 (Bio-Rad) with a user-defined threshold. Averaged triplicate HIV DNA measurements were normalized to one million PBMC with concurrent triplicate *CCR5* measurements [52] and LTR to internal HIV DNA target ratio were calculated per run. The range in copy numbers was typically ≤ 2-fold in replicates of triplicate determinations [52,69]. HIV DNA copy number was then normalized per one million CD4+T cells using %CD45%CD3%CD4 measurements obtained by flow cytometry [52].

### 2.6. Integration Site Identification

Integration site analysis was performed as previously described [16]. Briefly, DNA was isolated from Ficoll-purified PBMC (5–10 million), or FACS sorted T cell subsets. Total DNA was sheared into 300- to 500-basepair fragments with a Covaris M220 Focused-ultrasonicator (Covaris, Woburn, MA, USA). The human genomic regions and the linked viral sequences from both the 5′ and 3′ LTRs were amplified with linker-mediated nested PCR followed by paired-end sequencing using the MiSEQ 2×150-bp paired-end kit (Illumina, San Diego, CA, USA). The sequences of the host-viral junctions and the host DNA breakpoints were determined with a bioinformatics pipeline in which a stringent filter was used to ensure the quality of the integration sites recovered and the host DNA sequences were mapped to human genome (hg19) [16]. Sequences with identical integration sites but different host DNA breakpoints come from different cells, denoting proviruses that came from clonally expanded cells [16]. The number of clones and percent of integration sites belonging to clonal populations were determined with an in-house R script.

### 2.7. Characterizing Proviral Structure

To map the structure of individual proviruses, primer sets were designed based on sequences in the human genome near the integration site (Figure S6). Primers spanning different internal regions of the HIV-1 genome were tested using a nested PCR strategy that was performed with Phusion hot start flex polymerase (NEB, Ipswich, MA, USA), following the manufacturer’s instructions. Annealing temperatures were calculated with the NEB Tm calculator available online (tmcalculator.neb.com). The full length *HORMAD2* provirus (~700 basepair product) was PCR amplified with nested primers; host-specific forward primers complementary to a region in *HORMAD2* upstream of the site of proviral integration (Table S5; HORMAD2FO and HORMAD2FN) were combined with reverse primers that overlap the LTR U5 regions and the host genome at the integration site (HORMAD2 ol RO and HORMAD2 ol RN) (Figure S6A). PCR products were isolated by gel electrophoreses and Sanger sequencing was performed. Contiguous sequencing of PCR product confirmed that the proviral genome contained only a single LTR and no internal genes. Two nested PCR strategies to amplify internal HIV genes (*gag* or *nef)* combined with HIV/Host junction primers failed to amplify a product, indicating this provirus does not contain internal HIV genes (Figure S6B). Additionally, 3 μg of genomic DNA was sheared to 1500 basepairs using a Covaris Sonicator (Covaris). Sheared and non-sheared DNA was serially diluted 1:3 to an end-point and two rounds of PCR were performed as described above to ensure that the amplification of the solo LTR was not due to a PCR recombination artifact (Figure S6C). Nested PCR primer sequences used to amplify the solo LTR in *HORMAD2* are listed in Table S5.

### 2.8. Statistics

Linear regressions, correlations, one-way ANOVA with Tukey HSD correction for multiple comparisons, one sample *t*-tests, paired t-tests, and Fisher’s exact tests were performed in GraphPad, Prism version 7.

### 2.9. Data Sharing

The *HORMAD2* solo HIV LTR sequence has been deposited in the GenBank database (accession number: MN953428). The HIV integration sites reported here can be found on the Retrovirus Integration Database (RID, https://rid.ncifcrf.gov) [70].

## 3. Results

### 3.1. Participants

Samples were collected from 11 HIV-1 infected, ART-naïve participants who initiated cART as part of a natural history study of HIV infection at the NIH Clinical Center, Bethesda, MD. The participants had long-term suppression of viremia with HIV RNA levels below the detection limits of commercial assays (Table S1). Three groups were studied: 1) individuals initiating cART shortly after HIV infection (*n* = 4, AVBIO2_04, AVBIO2_07, AVBIO2_14, AVBIO2_26), all of whom had a documented history showing that they were infected for less than one year when cART was initiated, 2) individuals initiating cART during chronic HIV infection; (*n* = 5, AVBIO1_116, AVBIO2_05, AVBIO2_11, AVBIO2_17, AVBIO2_19,) all of whom had established HIV infection and evidence of CD4 lymphopenia (median CD4 = 373 cells/mm^3^; range 258–687 cells/mm^3^) and 3) individuals initiating cART after the onset of AIDS (*n* = 2, AVBIO2_08 and AVBIO2_21); (CD4+ T cell count below 200 cells/mm^3^). Individuals were chosen because they had undergone prolonged cART (median of 14.9 years, range: 6–16.4 years). During the course of long-term treatment, several participants underwent treatment interruptions due to personal preferences or as part of co-enrollment in early treatment interruption studies. In these participants, viremia was promptly resuppressed when cART resumed and suppression was maintained for a median of 9.3 years (range: 5.3–12.5 years).

### 3.2. HIV-1 DNA Quantification with Multiplexed Droplet Digital PCR Assays

We used ddPCR to quantify the absolute number of cells in blood that contained HIV-1 DNA prior to the initiation of treatment [52]. Before cART was initiated, HIV-1 DNA was detectable in samples from all participants. As shown in Table S2, there was an overall median of 3700 HIV-1 LTR DNA copies and 1200 HIV-1 *gag* DNA copies/10^6^ PBMC. In both cases, the range in copy number was >5-fold (1500–8300 for HIV-1 LTR DNA copies/10^6^ PBMC and 720–3700 HIV-1 *gag* DNA copies/10^6^ PBMC; Table S2). The reason(s) for this variation in viral DNA load prior to therapy, which was detected in all three groups of patients, is uncertain, but similar variation has been reported previously [51].

### 3.3. Loss of LTR and Internal HIV-1 DNA Sequences after Treatment Initiation

**Figure 1:**
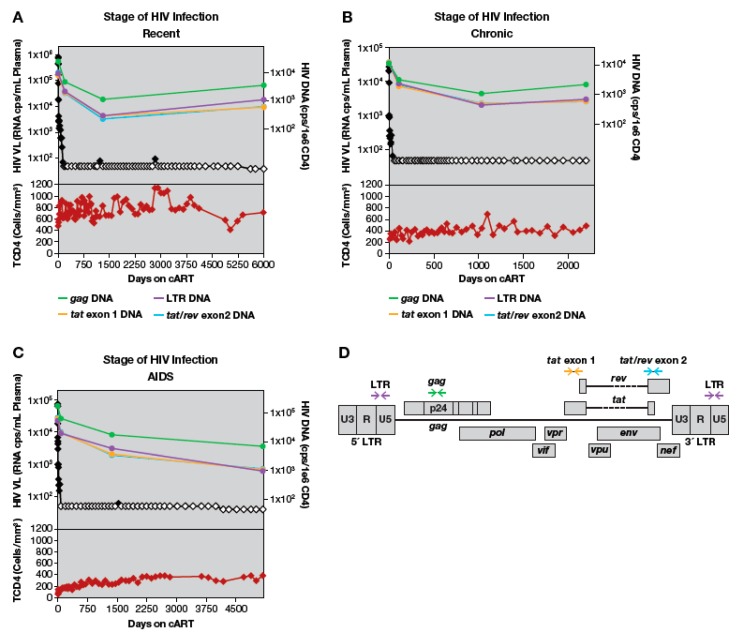
All HIV-1 DNA regions analyzed decline when combination antiretroviral therapy (cART) is initiated. HIV-1 DNA copies per 1 million CD4+ T-cells shown in green (long terminal repeat (LTR)), purple (*gag*), gold (*tat* exon 1), and blue (*tat/rev* exon 2). HIV viral load as HIV RNA copies per mL plasma is shown in black; open symbols represent values that are below the limit of detection. Total CD4 count in cells/mL shown in lower panel in red. Grey background represents periods of cART. (**A**) A participant treated during recent HIV infection AVBIO2_14. (**B**) A participant treated during chronic HIV infection, AVBIO2_17. (**C**) A participant treated during AIDS, AVBIO2_08. Error bars for copy numbers are omitted for clarity; as described in Methods, range in copy number was ≤2-fold in multiple determinations of each sample. (**D**). HIV-1 provirus map denoting positions of primers amplifying LTR, *gag*, *tat* exon1, and *tat/rev* exon2.

During the course of untreated HIV infection, infected cells can be created, persist, proliferate, or be eliminated, while maintaining a steady state of infected cells and viremia [71]. cART perturbs this steady state by preventing new infections, while allowing the other processes to continue. Concurrently, there is a degree of immune reconstitution following the introduction of cART, with an increase in the number of CD4+ T cells in the blood. To investigate changes in the levels of HIV-1 DNA-containing cells following initiation of cART, we quantified HIV-1 LTR and *gag* DNA copy numbers in peripheral blood prior to the introduction of cART, at the time of viral suppression (< 50 copies HIV-1 RNA/mL plasma, median 82 days of cART), during third phase viral decline (range: 1.8 to 4.4 years on cART) and after prolonged therapy (median 13.1 years on cART) (Figure 1 and Figures S1 and S2). We used multiplexed ddPCR assays to quantify the absolute number of cells in blood that contained HIV-1 LTRs as well as those that contained internal HIV-1 DNA targets [52]. Populations of peripheral lymphocytes undergo profound changes after individuals initiate cART, and the increase in the number of CD4+ T cells may include cells with HIV proviruses. To investigate dynamics of HIV proviral landscape in detail, we calculated the numbers of proviruses both as the concentration of total and deleted proviruses in blood (HIV-1 DNA/mL blood) and as the fraction of HIV-infected cells in the CD4+ T cell population (HIV-1 DNA/10^6^ CD4+ T cells) (Figure S3). This approach allowed us to quantify the absolute number of HIV proviruses, even if their frequency declined during therapy.

In the majority of patients, the absolute numbers of HIV-1 DNA copies and the fraction of HIV-infected cells (proviruses/10^6^ CD4+ T cells) had declined by the time viral RNA levels in plasma had decreased to <50 copies/mL, but the viral DNA declined to a much lower extent (Figure 1, Figure S1). HIV-1 DNA LTR copy numbers/10^6^ CD4+ T cells declined a median of 6.2-fold (range 2 to 25-fold) compared to pretherapy values (Figure 1, Figure S1). Similarly, the fraction of cells that contained HIV-1 LTR sequences declined a median of 2.9-fold compared to pre-therapy (range: 1 to 4.3-fold), and the fraction containing internal HIV-1 DNA sequences declined 2.6-fold (range: 1.4 to 4.8-fold) (Figure S2). Overall, the kinetics of decay were similar when HIV copy numbers were expressed as proviral copies/mL blood (Figure S3). These data demonstrate that, upon initiating cART, HIV-infected cells decline both in absolute number and as proportion of CD4+ T cells in the blood.

In a single patient (AVBIO2_21), total HIV-1 DNA copy numbers increased 2.5-fold at the time viral RNA levels had declined to <50 copies/mL plasma, while the fraction of HIV-infected cells declined (Figure S3). In this study participant, there was a large increase in the number of CD4+ T cells during the initial period on cART. Even though a relatively small fraction of these cells were infected, the overall recovery of the CD4+ T cells led to an increase in the number of infected cells in the blood (see below for detailed analysis).

After the initial decreases in HIV-1 DNA during the first 3–4 years on cART, we did not detect any additional decreases in proviral copy number in any of the individuals studied, regardless of their baseline recent, chronic, or HIV/AIDS status (Figure S3). After a median of 13.1 years on cART, the fraction of infected cells, measured by median HIV-1 LTR DNA levels, was maintained at 920 copies/10^6^ PBMC (range 210–4500) and the levels of internal HIV-1 DNA sequences, was, on average, 200 copies/10^6^ PBMC (range 90–730) (Table S2). These data suggest that, for the majority of patients, the largest changes in the concentration of HIV-1 DNA-containing cells occurs in the first 1–3 years after cART is initiated. 

### 3.4. Quantitation of HIV-1 DNA LTR and Internal Sequences Using Multiplexed ddPCR

The data presented above show that the numbers of HIV proviruses declined in most patients after treatment was initiated but remained relatively stable during prolonged cART. To determine if there was preferential loss, over time, of cells that contained certain types of proviruses (for example, proviruses containing *gag* or *tat/rev* versus highly defective/deleted proviruses), we used multiplexed ddPCR to simultaneously measure levels of HIV-1 LTR and internal DNA sequences [52]. The ratio of LTR to internal HIV-1 DNA sequences (*gag, tat* exon 1, and *tat/rev* exon 2) reflects the overall composition of the proviruses, specifically the relative proportions of proviruses with internal deletions and/or APOBEC-mediated hypermutations that would affect the detection of the targeted sequences. An intact provirus contains two LTRs and one copy each of sequences detected by the *gag, tat* exon 1, and *tat/rev* exon 2 primer sets. Thus, a ratio of HIV-1 LTR sequences to internal DNA sequences that is greater than 2 indicates that internally deleted and/or hypermutated proviruses are present, while ratios approximating 2 imply that the majority of the proviruses in the population are not deleted or hypermutated in the internal regions we assayed. We optimized our assays using cell-associated DNA extracted from either uninfected CEM cells or ACH2 cells, an HIV-infected cell line that has been reported to harbor, principally, a single intact replication-competent provirus [72,73]. As described (Methods, Figure S4), ddPCR quantified HIV proviruses in ACH2 cells at a level of 1.2 proviruses/ACH2 cell, which is consistent with recent studies demonstrating that these cells have low level ongoing HIV replication [60,61], with a slow accumulation of additional proviruses. The LTR:*gag* ratio was (1.8 +/− 0.19), slightly, but statistically significantly less (t-test, *p* = 0.0001) than the 2:1 ratio expected from intact proviruses. As described in Methods, the decreased number of LTRs may be due to aberrant proviruses, incomplete products of reverse transcription, and one-LTR circles, which are readily detected in ACH2 cells [62]. Only a few false positive droplets (< 1 copy/60,000 cells) were detected in analyses of uninfected CEM cells (Supplemental Figure S4A, CEM). 

### 3.5. Proportion of Deleted and/or Highly Defective Proviruses Increases after Long-Term cART

**Figure 2:**
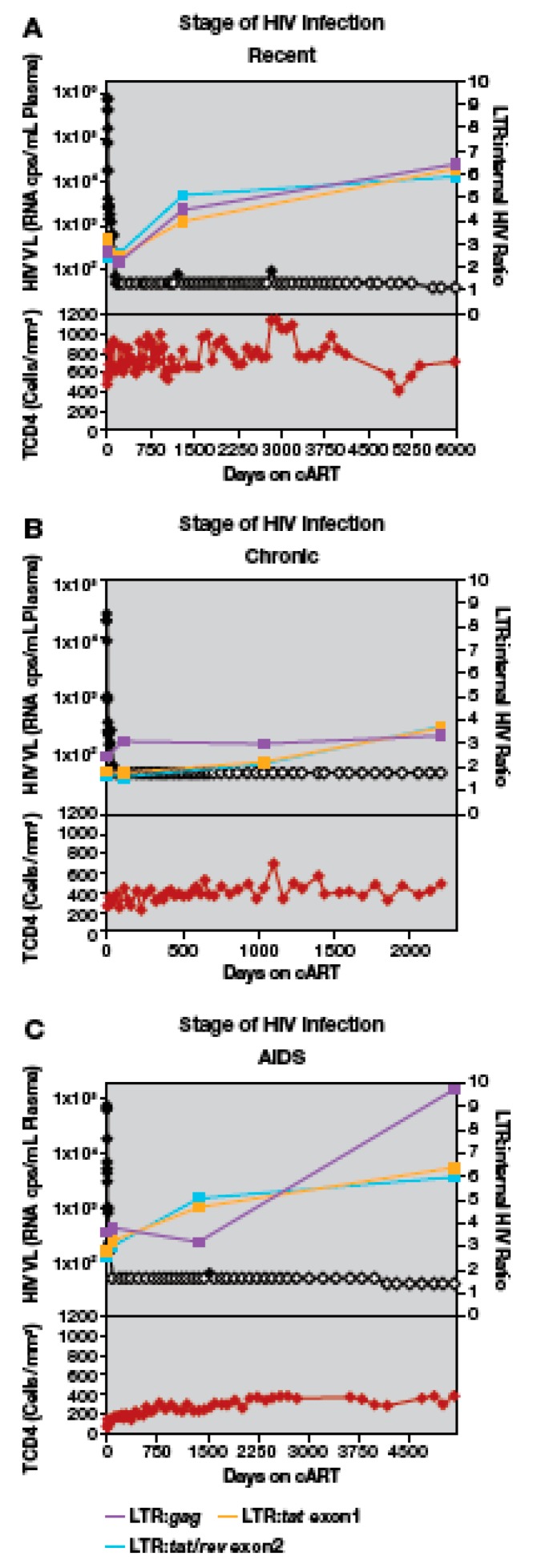
The ratio of HIV-1 LTR DNA to internal HIV-1 DNA regions during cART. HIV viral load (HIV-1 RNA copies per ml plasma) is shown in black; open symbols represent values that are below the limit of detection. LTR:*gag* ratios (purple), LTR:*tat* exon 1 ratios (gold), LTR:*tat/rev* exon 2 ratios (blue) are also shown. Total CD4 counts (cells/mm^3^) are shown in the lower panel in red. Grey background represents periods of cART. (**A**) A participant treated during recent HIV infection AVBIO2_14. (**B**) A participant treated during chronic HIV infection, AVBIO2_17. (**C**) A participant treated during AIDS, AVBIO2_08.

**Figure 3:**
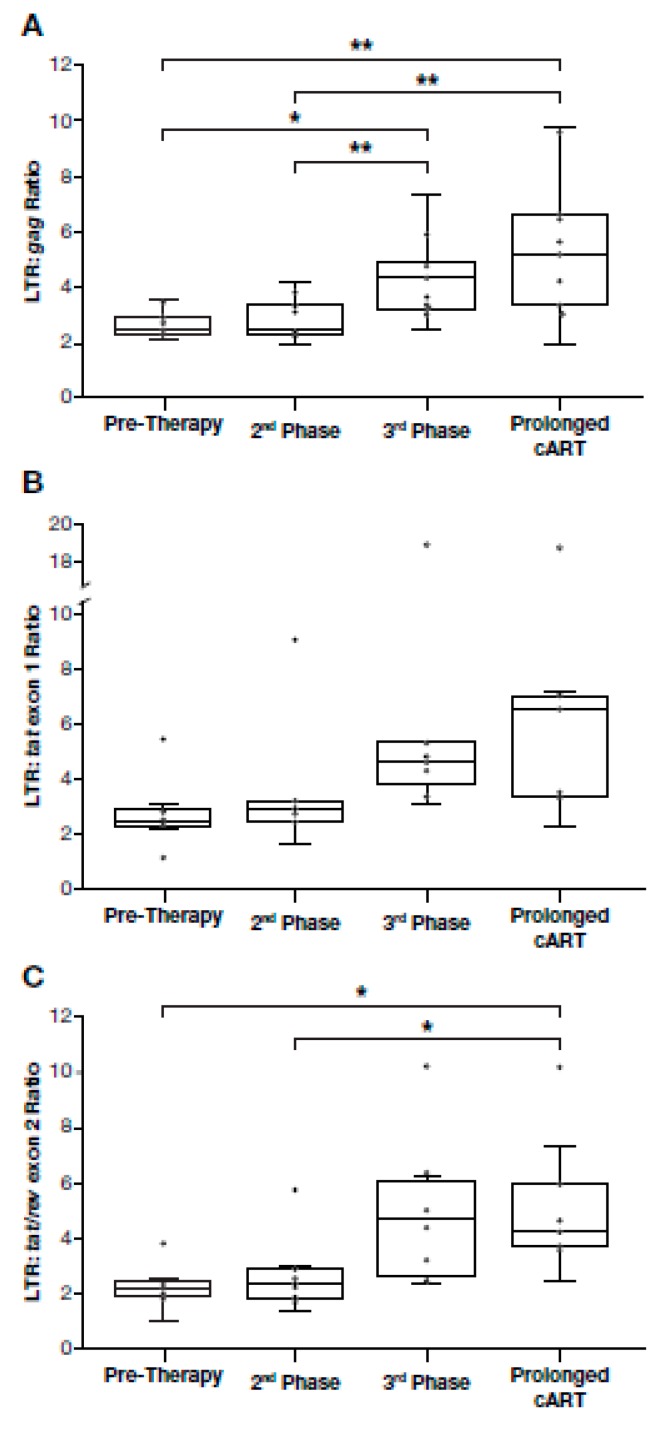
HIV LTR to internal HIV-1 DNA target ratios increase in most individuals after prolonged cART. Box and whisker plots of HIV-1 LTR DNA to internal HIV-1 DNA ratios for all study participants were determined at pretherapy, after viral suppression, during 3^rd^ phase viral decay, and by prolonged cART (one-way ANOVA, Tukey HSD *p* < 0.05, ** *p* < 0.01; outlier values included as indicated; all other pairwise comparisons were not significant, LTR:*tat* exon 1 *p* = 0.59, and LTR:*tat/rev* exon 2 *p* = 0.58). (**A**) HIV-1 LTR DNA to HIV-1 *gag* DNA ratios, (**B**) HIV-1 LTR DNA to HIV-1 *tat* exon 1 DNA ratios and (**C**) HIV-1 LTR DNA to HIV-1 *tat/rev* exon 2 DNA ratios.

To investigate the extent to which the populations of defective proviruses change during cART, we calculated LTR to internal HIV-1 DNA ratios for each timepoint analyzed in our cohort (Figure 2, Figure S5). In untreated individuals there are ongoing rounds of virus replication and most cells that have recently been infected with HIV die rapidly; thus, we expected that the majority of the HIV-1 DNA in pre-therapy samples was the result of recent infection. In a cumulative analysis of data from all the study participants (Figure 3), the overall median ratio of LTR DNA to *gag* DNA in the pre-therapy samples was 2.4 (range: 2.1–3.6), a value that is significantly greater than 2 (one sample t-test *p* = 0.019). Elevated LTR:*gag* DNA ratios were detected in all patients in all groups, suggesting that proviruses with internal deletions and/or hypermutations are generated and accumulate during active cycles of replication [14]. Similar increases were measured in comparisons of LTR:*tat* exon 1 and LTR:*tat/rev* exon2 (Figure 2, Figure S5). The median ratio of LTR to *tat* exon 1 sequences during pre-therapy was 2.4 (range: 1.2–5.4) from the nine participants for whom *tat* exon 1 sequences amplified, and the median ratio of LTR to *tat/rev* exon 2 was 2.2 (range 1–3.8). There was more variation in the latter ratios, however, and they were not significantly different from 2:1 (two-sided t-test *p* = 0.12, and *p* = 0.41 respectively).

We performed an analysis of the amounts of HIV-1 DNA present during cART in all study participants. After initial first and second phase decay of viremia (median: 82 days), there was no change in the ratios of LTR to internal sequences in PBMC compared with pre-therapy samples (Figure 3). Overall, these results indicate that, in the majority of participants, there was no enrichment for HIV-1 DNA with large deletions and/or hypermutations soon after most of the short-lived virus-producing cells were lost. One participant (AVBIO2_21), with the highest ratios of LTR to internal HIV-1 sequences (Figure 3), had a large expansion of a single clone (see below).

In contrast to what we detected early after the initiation of cART, the ratio of LTR DNA:internal HIV-1 DNA increased significantly during prolonged cART in most individuals. After a median of 3.7 years on cART (3rd phase), *gag* sequences declined significantly relative to LTR sequences (Figure 3) (median LTR:*gag* ratio*:* 3.8, range: 2.4–7.7, *p* = 0.033). Increases were detected in the ratios of LTR:*tat* exon 1 (median LTR:*tat* exon 1 ratio*:* 4.4, range: 1.9–5.3) and LTR:*tat/rev* exon 2 (median LTR:*tat/rev* exon 2 ratio*:* 4.4, range: 2.4–6.4), but the increases were not statistically significant at 3.7 years on cART (*p* = 0.32 and *p* = 0.051, respectively) (Figure 3). The increase in the LTR:internal sequences ratio ranged from 1.7-fold (*gag*) to 2.3-fold (*tat/rev* exon 2; Figure 3), and increases were detected in the majority of individuals, including those who initiated cART with recent or chronic HIV infection or individuals with AIDS. One participant with AIDS did have consistently higher ratios of LTR:internal HIV-1 sequences (Figure 3, AVBIO2_21, outlier, see below for detailed analysis). Taken together, these data indicate that there is enrichment of deleted proviruses in individuals who initiated cART regardless of the level of baseline HIV-associated immunodeficiency. During successful cART, HIV-1 DNA with deletions and/or hypermutations affecting the internal PCR assays come to dominate the population, but only after first and second phase decline in the viral load. 

In all participants but one, for whom cART was maintained for a much longer time (median of 13.1 years, range: 5.3–16.4 years), the on-therapy HIV-1 LTR DNA to internal HIV-1 DNA ratios increased compared to pre-therapy (Figure 3). After prolonged cART, the median ratio of LTR to *gag* was 5.2 (range: 1.9–9.7), the median ratio of LTR to *tat* exon 1 was 5.0 (range: 2.3–7.1), and the median ratio of LTR to *tat/rev* exon 2 was 4.0 (range: 2.4–7.4) (Figure 3). The increases in LTR:*gag* and LTR*:tat/rev* exon 2 ratio*s* over time were statistically significant (LTR:*gag p* = 0.010; LTR:*tat/rev* exon 2 *p* = 0.011) (Figure 3); increases in LTR:*tat* exon 1 ratios were detected, but were more variable than LTR:*gag* or LTR:*tat/rev* exon 2, likely due to sequence variation in *tat* exon 1 (see Methods) and LTR:*tat* exon 1 increases were not statistically significant (*p* = 0.13). Collectively, these findings indicate that, during prolonged cART, there is an increase in the proportion of deleted/highly defective proviruses in the majority of individuals who initiated cART with recent or chronic HIV, or in individuals with AIDS.

Although there was an increase in LTR:*gag* sequence ratios in most of the donors, there were a few individuals for whom the LTR:*gag* sequence ratio remained approximately 2:1 (Figure 2 and Figure S5; participants AVBIO2_04, _05, _17). The reasons for this result are not known; these donors included one individual who started cART early after infection and two who had prolonged infection prior to cART. In the other 8 participants, there was at least a 1.8-fold increase in LTR to *gag* DNA ratios from pre-therapy to long-term cART (range: 1.8 to 4.8-fold increase) (Figure 2 and Figure S5). Analysis of viral DNA in cells from two of the participants (AVBIO2_05 and 17) who did not show an increase in LTR:*gag* ratios after prolonged cART showed a 2-fold increase in the LTR:*tat* exon 1 or LTR:*tat/rev* exon 2 ratios compared to pre-therapy. Samples from the remaining participant (AVBIO2_04) were below the limit of detection for both *tat* and *rev*, presumably due to primer mismatches (Figure S5). These data suggest that extensively deleted/highly defective proviruses do not dominate the population of HIV-infected cells in all individuals.

### 3.6. Dynamics of HIV Proviral Populations after Treatment Interruption

**Figure 4:**
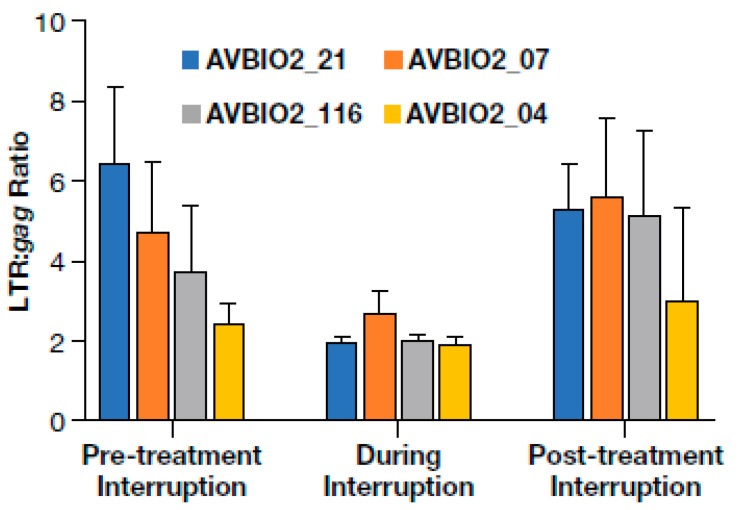
Cells containing deleted proviruses are present after treatment interruption and resuppression. The effects of treatment interruption and subsequent resuppression on HIV-1 LTR and *gag* ratios in four individuals who experienced treatment discontinuation. LTR DNA to *gag* DNA ratios before, during, and after treatment interruption. Error bars represent the standard deviation of ratios calculated from 3 to 12 replicates.

In this study, we investigated samples from individuals undergoing cART for prolonged periods. During the course of their treatment, four individuals discontinued therapy either due to personal reasons or as part of the study design. Since analytical treatment interruptions have become an important way to assess curative strategies, it was of interest to quantify proviral populations during and following these interruptions to shed light on the dynamic changes resulting from cART discontinuation. In most patients, if cART is discontinued, viremia rebounds to near pre-therapy levels within weeks [8,9,10] with virus that is genetically similar to the pre-therapy virus [74]. When cART was restarted in participants who had undergone short treatment interruptions, the number of HIV-1 LTR DNA copies returned to levels comparable to those present prior to the treatment [75,76]. The effects of treatment interruption on the proportion of deleted proviruses has not been investigated, however, and we studied samples from individuals in our study who interrupted therapy either from nonadherence (AVBIO2_21 and _07, range 1–3 periods of treatment discontinuation lasting a median 320 days each) or as part of a treatment study that included multiple structured treatment interruptions (AVBIO2_116 and _04, range 5–8 periods of treatment discontinuation lasting a median of 28 days each). To investigate the effects of these treatment interruptions on the proviral landscape, we determined the LTR to *gag* ratios before, during, and after the periods when cART was discontinued (Figure 4).

Prior to treatment interruption, the participants had been suppressed on cART for a median of 3.3 years (range: 2–7 years) and the average LTR:*gag* ratio was 5.2 (range: 2.5–6.5). During treatment interruptions, viremia increased by a median of > 50,000-fold, with an increase of cell-associated HIV-1 DNA of approximately 22-fold and a decrease in the LTR:*gag* ratio that averaged 2.2-fold (range: 1.8 to 3.5-fold). This change presumably reflects the effects of adding newly infected cells, many of which carried non-deleted proviruses, to the relatively stable population of largely defective proviruses that persist on therapy. After cART was reinitiated and the participants were continually suppressed for a median of 7 years (range: 1.5–12 years), the LTR:*gag* ratio increased an average of 4.8-fold (range: 3.0 to 5.6-fold). These values are not statistically different from the LTR:*gag* ratio before treatment interruption (paired t-test *p* = 0.12). Our findings imply, as expected, that the non-deleted proviruses that were newly acquired during the treatment interruption were preferentially lost after cART was reinitiated and that the deleted/highly defective proviruses were maintained during multiple prolonged treatment interruptions and resupression on cART.

### 3.7. Changes in the Size of a Large HIV-Infected Clone

The mechanisms that underlie the observed increases in the ratio of LTR to internal HIV-1 DNA sequences are not known. It is likely that cells that harbor largely intact proviruses and express viral RNA are preferentially lost due to the toxicity of the viral proteins, immune surveillance by the host, or both [26,27]. There could also be preferential clonal expansion of individual HIV-infected cells that carry deleted/highly defective relative to cells that carry largely intact proviruses. The emergence, subsequent expansion, and persistence of clones is not well understood. To learn more about the role clonal expansion can play in shaping the population of HIV-infected cells, we obtained samples from a participant who had clones of HIV-infected cells for which we had previously determined the integration sites [16]. Because the kinetics of clonal expansion of individual HIV-infected cells during cART have not been described, we developed ddPCR assays to quantify the proviruses in specific clones of HIV-infected cells.

We characterized a large HIV-infected cell clone with the provirus integrated in the *HORMAD2* gene identified in study participant AVBIO2_21. As noted above, this study participant showed an increase in HIV proviruses during cART and had the highest ratios of LTR:*gag,* LTR:*tat* exon 1, and LTR:*tat/rev* exon 2 detected in any group on prolonged cART (Figure 3). The *HORMAD2* clone was the largest HIV-infected clone, present at a frequency of c. 20%, as previously reported [16], and it was the largest clone seen in this study. The integration site assay showed that the provirus was integrated at position 30,515,408 (hg19) of chromosome 22, in the opposite orientation relative to the *HORMAD2* gene [16]. Multiple combinations of overlapping host-HIV primers (Figure S6) in limiting-dilution PCR were used to determine that the *HORMAD2* provirus consists of a solo HIV LTR, which was confirmed by sequencing the whole insert from host junction to host junction. Solo LTRs are created by homologous recombination between the LTRs at each end of the provirus, and have been described for other retroviruses and LTR retrotransposons [77,78] but, to date, they have not been described for HIV. We found the solo LTR in *HORMAD2* was present in total PBMCs prior to the initiation of cART, but as a smaller fraction of the proviruses present (Figure S6A).

**Figure 5:**
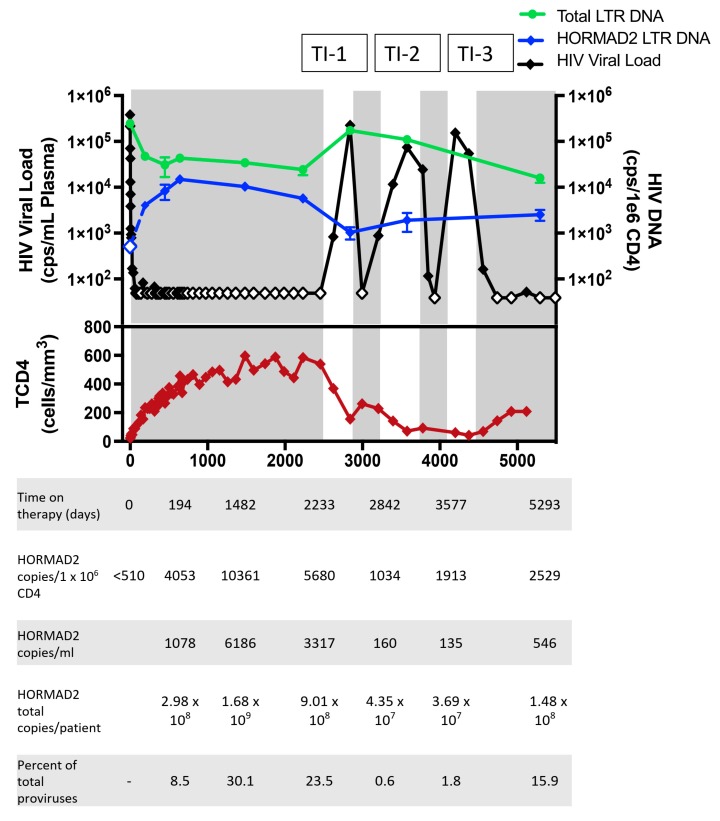
Dynamics of the clonal expansion of cells harboring a solo LTR integrated in *HORMAD2.* Abundance of the solo LTR provirus in the *HORMAD2* gene in AVBIO2_21 over time measured with the Droplet Digitial PCR (ddPCR) integration site specific assay (Figure S6). HIV viral load (black diamonds), HIV-1 LTR DNA copies per million CD4+ T cells (green circles), *HORMAD2* provirus copies per 1 million CD4+ T cells (blue diamonds), total CD4 count (red diamonds) are shown in the lower panel. Shaded areas indicate periods of cART, blank areas periods of treatment interruption (labeled TI-1, -2,-3). Open symbols indicate values that are less than the limit of detection.

To investigate the relative levels of the HIV-infected cell clone with the provirus in *HORMAD2*, we developed a specific ddPCR assay using a forward primer that overlaps the HIV/host junction at the *HORMAD2* integration site and an HIV-1 LTR-specific reverse primer and probe (Figure S6D). In the pre-therapy samples the *HORMAD2* solo LTR was detectable by a bulk PCR (Figure S6A), but was below the limit of detection of our ddPCR provirus-specific assay (<500 copies/10^6^ CD4+ T cells) or approximately <0.2% of LTRs (Figure 5). By day 194 of cART, the *HORMAD2* provirus was detectable using the specific ddPCR assay at 1080 copies/mL which, if the distribution of this clone is comparable to the distribution of total CD4 T cells, corresponds to approximately 3 × 10^8^ total copies in the patient. At this time, the abundance of the *HORMAD2* provirus had increased by at least 8-fold compared to pre-therapy, comprising 8.5% of the LTRs measured. After 4 years on therapy, the *HORMAD2* provirus was present at 6190 copies/mL (estimated 1.7 × 10^9^ copies in the patient; 10,400 copies/10^6^ CD4+ T cells) but subsequently declined 1.8-fold to 3320 copies/mL after another 2 years on cART. At this point the clone still comprised 23% of the LTRs and was present as ~9 × 10^8^ copies in the patient (Figure 5). Thus, the cell clone harboring the *HORMAD2* provirus underwent a rapid expansion shortly after treatment was initiated and, despite a moderate decline in the size of the clone, the clone was maintained for at least 6 years on suppressive antiretroviral therapy.

The study participant with the solo LTR in *HORMAD2* underwent multiple periods of treatment nonadherence, but viremia was resuppressed each time cART was re-initiated (Figure 5). To study the effects of treatment discontinuation on the clone we investigated the abundance of the *HORMAD2* provirus before, during, and after multiple treatment discontinuations. During the treatment interruptions, HIV-1 DNA levels increased. HIV-1 LTR copies/10^6^ CD4+ T cells increased 6-fold (141,000 copies/10^6^ CD4+ T cells, t-test *p* < 0.001), and the copies of HIV-1 *gag* increased 18-fold (72,000 copies/10^6^ CD4+ T cells, t-test *p* < 0.001). After 6 months of cART discontinuation the total CD4 count decreased 8-fold (Figure 5). During the first treatment interruption, the *HORMAD2* provirus remained detectable at 160 copies/mL (4.3 × 10^7^ copies in the body, a 20-fold decrease compared to the timepoint prior to the treatment interruption). Because the number of HIV-infected cells had increased, the clone accounted for only 0.6% of the LTRs. One year later, during a second interruption, the *HORMAD2* provirus was present at 135 copies/mL or 3.7 × 10^7^ total copies in the body (Figure 5). These findings indicate that the size of the expanded cell clone with the *HORMAD2* solo LTR declined, but the clone survived during a period of ongoing replication and substantial CD4+ T cell death. Two years after resuppression, the *HORMAD2* provirus was present at 550 copies/mL (1.5 × 10^8^ copies of the *HORMAD2* clone in the patient) (Figure 5). While the size of the *HORMAD2* clone declined relative to total CD4+ cells, it remained detectable during multiple treatment interruptions and resupression (Figure 5), suggesting that a persistent immune response might have caused its maintenance.

### 3.8. The HORMAD2 Clone is Enriched in Effector Memory Cells

**Figure 6:**
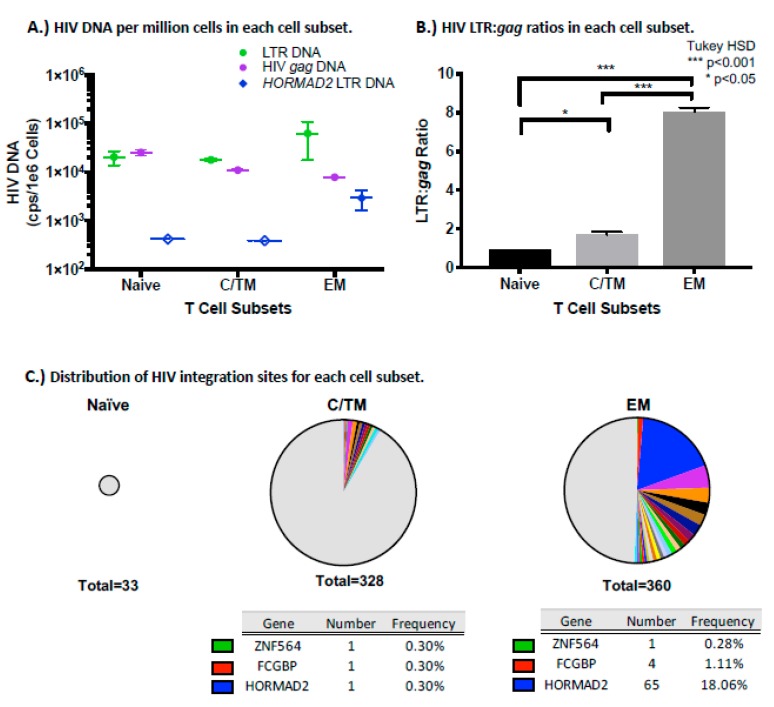
An expanded cell clone harboring a solo LTR in *HORMAD2* is enriched in the effector memory subset. Sorted T cell subset analysis showed that the *HORMAD2* provirus is primarily in the effector memory subset of participant AVBIO2_ 21: **A**) Frequency of the LTR (green circles) and *gag* (purple circles) DNA and the expanded cell clone with the provirus in *HORMAD2* (blue diamonds) in the three T cell subsets. Dashed bars and open symbols indicate the limit of detection. **B**) HIV-1 LTR to *gag* DNA ratios in the sorted cells (one-way ANOVA, Tukey HSD * *p* < 0.05, *** *p* < 0.001). **C**) Pie charts of integration sites obtained by ISA for cell subsets. The size of the chart reflects the absolute number of integration sites recovered. Integration sites obtained only once are indicated in the grey segment, while colored portions represent integration sites identified more than once (cell clones). Tables list the frequency of integration sites that were found in both the CTM and EM T cell subsets.

The differentiation of memory T cells occurs in a stepwise fashion towards progressive commitment to more differentiated cell types [79]. When naïve T cells recognize their cognate antigen, they undergo clonal expansion and differentiate into central memory (CM) and effector memory (EM) cells [79,80,81]. CM T-cells have longer half-lives and higher proliferative capacity compared to EM [79]. An intermediate subset, transitional memory (TM), is in a maturation state between CM and EM [82]. To determine what types of T cells carried the *HORMAD2* provirus, we sorted total PBMC from participant AVBIO2_21 after 2625 days on study, when the viral load had risen to 830 HIV-1 RNA copies/mL shortly after a treatment discontinuation (Figure 5). The different subsets were identified based on the distribution of the CD27 and CD45RO cell surface markers, as described (55). First, HIV-1 LTR and *gag* DNA were quantified by ddPCR from naïve, a mixture of central and transitional memory (CTM), and effector memory (EM) T-cells and LTR:*gag* DNA ratios were calculated to determine which CD4+ T cell subset harbored the most defective/deleted proviruses in this individual (Figure 6). All of the T cell subsets we analyzed had comparable amounts of HIV-1 LTR DNA (range: 18,000–37,000 copies/10^6^ cells, *p* = 0.14), but HIV-1 *gag* copies varied among the cell subsets (*p* = 0.006).

Proviruses were detected in the naïve cell subset, but it is not clear from this analysis whether these proviruses are in cells that are truly naïve or in T stem cell memory cells, which are also CD27+CD45RO- and will be present in this subset. The naïve cell subset had the highest concentration of HIV-1 *gag* at 25,000 copies/10^6^ cells, but the yield of naïve cells was low in this patient, and the total number of proviruses was less than that detected in CTM or EM. In the naive T cell subset, the LTR:gag ratio was <2. As noted above, LTR:*gag* ratios < 2 may be due to the presence of proviruses with aberrant integrations that are missing either the 5′ or 3′ LTR. Previously, Heiner et al. reported that intact proviruses are enriched in the EM CD4+ T cell subset [83]. However, in the one patient we studied here, we found fewer copies of HIV-1 *gag* DNA in EM compared to CTM (4600 vs 11,000 copies/10^6^ cells). Thus, the EM cells had the highest LTR to *gag* ratio, 8.0 (Figure 6B), reflecting a significant enrichment for deleted/highly defective proviruses, consistent with the relatively high LTR:*gag* ratio in the unfractionated PBMC from this study participant (Figure S5, AVBIO2_21). The CTM subset, in contrast, had LTR:*gag* ratio of c. 2, suggesting no enrichment for deleted proviruses. Taking into account the yield of cells from the sorting procedure, we found that the majority (59%) of LTR DNA was in the EM subset, but this subset also had the smallest fraction (25%) of total *gag* DNA from this participant. Using the *HORMAD2* provirus-specific ddPCR assay, we were able to detect the *HORMAD2* solo LTR in the EM subset (2900 copies/10^6^ cells) (Figure 6A); however, the *HORMAD2* solo LTR was below the limit of detection of the assay in both naïve (<410 copies/10^6^ cells) and CTM subsets (<380 copies/10^6^ cells).

CM can proliferate and differentiate into EM when T cell receptor activation occurs and in response to cytokines [80,81]. To ask whether the high LTR:*gag* ratio in the EM subset reflected clonal expansion of HIV-infected cells, we recovered integration sites (IS) from the sorted subsets (Figure 6C). No clones of HIV-infected cells were identified in the naïve T cell subset, although only 33 total integration sites were recovered from this subset. While similar numbers of integration sites were recovered in the EM and CTM, over half of the HIV-infected cells in the EM subset were shown to be in clonally expanded cells (181/360 IS within 24 clones) compared to CTM (25/328 IS within 11 clones) (Fisher’s exact test, *p* < 0.0001). The cell clone harboring the solo LTR in *HORMAD2* was much more prevalent in the EM subset (720 copies/mL) than in naïve or CTM subsets. It is, in fact, unclear if there really are any cells with the provirus in *HORMAD2* in the CTM subset; the few copies we detected could have come from EM contamination, even if the sort was >99.5% pure. Taken together, these findings confirm that the *HORMAD2* provirus is overrepresented in EM, suggesting that the cells that harbored the *HORMAD2* provirus could have expanded in response to antigen stimulation. As shown in Table S3, other proviruses with integration sites in FCGBP or ZNF564 were found in both CTM and EM but were not enriched in EM. There were additional clones that were detected in EM; several of the additional proviruses were in cells that were clonally expanded in the EM subset (integration sites in TSG101, TGFBR3, YPEL1, SLX4,CKAP5, and HELB), demonstrating that there were additional clones of HIV-infected cells in EM subset. 

## 4. Discussion

In individuals with established HIV infections, HIV populations are large and genetically diverse [53]. Upon initiating cART, viral RNA levels in plasma undergo profound decline during the first few weeks. In contrast to the large (>17,000-fold) decay in HIV viremia, others have demonstrated that the levels of cell-associated HIV-1 DNA decline about 1000-fold less (10 to 30-fold) after the first year of cART. Viral DNA continues to decline slowly in most individuals over years on suppressive therapy [23]. The majority of the proviruses that remain in those on successful therapy are defective (>98% by one estimate) [13,24]. Prior studies have investigated levels of HIV-1 DNA by amplifying HIV LTR or internal HIV sequences, but not both simultaneously, and the dynamics of population shifts are not well understood. To gain additional insight into the shifts in the proportion of defective proviruses we investigated cell-associated HIV-1 DNA levels in PBMC in individuals for prolonged periods on cART using a multiplexed ddPPCR approach to quantify total and defective proviruses.

Here, we show that total HIV-1 LTR DNA levels remain relatively stable during the first several years of cART and the ratio of LTR to internal HIV-1 DNA targets (including *gag* and *tat/rev* exons 1 and 2) increases over time in most of the individuals we analyzed, likely reflecting a loss of proviruses containing *gag* or *tat/rev*. As previously reported [14], highly deleted/defective proviruses are present during acute or chronic HIV infection, as well as in cells infected *ex vivo*, but, as we show here, highly deleted/defective proviruses most likely represent a much smaller proportion of total proviral population prior to cART when a substantial proportion of the cell associated HIV-1 DNA prior to cART is unintegrated. As revealed by the LTR:*gag* DNA ratio, deleted proviruses begin to predominate after first and second phase viral decay, after the majority (> 75%) of the HIV-infected cells have been eliminated. These data imply that proviruses containing *gag* or *tat/rev* are preferentially lost in most patients only after prolonged cART. Enrichment in the fraction of proviruses that were deleted occurred in all three groups of individuals studied, in individuals with recent or chronic HIV infection, or with advanced HIV/AIDS, suggesting enrichment of deleted proviruses occurred despite advanced immune deficiency present at the time cART is initiated.

**Figure 7:**
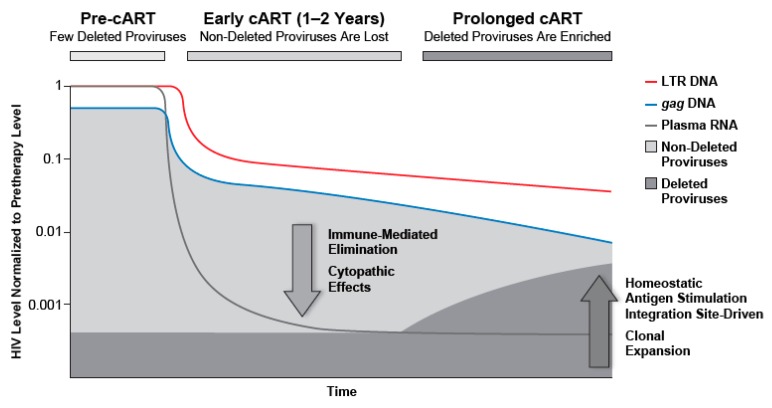
Proportion of proviruses that are deleted increases during cART. Prior to cART, non-deleted proviruses dominate the proviral landscape. After a period of viral suppression, defective viruses, including deleted and hypermutant proviruses are enriched; after prolonged cART, such proviruses dominate the proviral landscape.

The forces that drive the change in proviral composition but maintain the overall number of proviruses during cART are not well understood. Because there is no detectable active ongoing HIV replication during cART [1,2], the shift in the populations of HIV-infected cells reflects changes in the sizes of the various populations of infected cells, including clonal expansion of uninfected and infected T cells, and elimination of some of the infected T cells (Figure 7). Immune factors, which can persist in individuals who have advanced disease, are likely to play prominent roles. For example, homeostatic proliferation and antigen-induced expansion of specific CD4 clones likely contribute to the maintenance and the clonal outgrowth of at least some of the HIV-infected cells [83]. In our studies, the majority (10/11) of the study participants experienced decreases in the concentration of HIV-infected cells, expressed as copies/mL blood or copies/10^6^ CD4+ T cells. In one patient, who had a relatively low CD4+T cell count upon initiating cART and experienced a substantial increase in CD4+ T cells during cART, we detected an increase in the number of infected cells if the data are expressed as HIV-1 DNA copies/mL; however, this individual showed a decline in fraction of cells that were infected if the data were expressed as HIV-1 DNA copies/10^6^ CD4+ T cells. These findings suggest that the CD4+ T cell recovery can cause expansion of HIV-infected cells exceeding the overall loss of infected cells during cART. For the remainder of study participants, there was a decline in either the number of HIV-infected cells/mL or proviruses/CD4+ T cells.

The recovery of immune function after cART is initiated may contribute, not only to the clonal expansion, but also to the elimination of HIV-infected cells and could result in a preferential loss of infected cells that express HIV antigens. Selection against proviruses that contain *gag* or *tat/rev* sequences is consistent with our findings that HIV-1 proviruses containing *gag* and/or *tat/rev* are lost more rapidly than those that do not contain these HIV sequences. Although the majority of proviruses are defective or deleted in individuals on long-term cART [14,15] some defective proviruses are still potentially capable of producing peptides that can be displayed on the infected cell surface where they can be recognized by HIV-1 specific CTLs, at least in ex vivo transfection experiments [26,27]. The expression of viral antigens by a fraction of the cells containing deleted and defective proviruses could also contribute to persistent ongoing immune activation and dysregulation. The selective elimination of cells harboring proviruses that carry and express *gag* would shift the proviral landscape, leading to an increase in the fraction of proviruses that cannot express *gag* or that lack *gag*, which would contribute to an increase in the HIV-1 LTR:*gag* ratio. A new ddPCR assay, the intact provirus detection assay (IPDA), is now available that rapidly estimates the numbers of cells that carry what are likely to be infectious proviruses [45]. Our approach estimates total HIV-1 DNA by quantifying HIV-1 LTRs, enabling the analysis of differential decay kinetics between largely intact versus deleted/highly defective proviruses. Measuring the LTR:*gag* (or *tat*) ratio informs our understanding of the differential decay of genetically intact vs defective proviruses measured by the recently described IPDA assay and by standard quantitative virus recovery assay (QVOA) (44), supporting the idea that, even during long term ART, there is a slow but detectable loss of non-deleted proviruses and a relative enrichment of largely defective ones. Large studies analyzing total, deleted and infectious proviruses will provide a useful comparison between assays and a better understanding of the forces that shape the fate of infected cells and whether forms of persistent proviruses contribute to persistent immune activation.

The clonal expansion and/or subsequent loss of large clones of infected cells carrying deleted/highly defective proviruses can also result in a substantial shift in the population of infected cells. Cells harboring proviruses can undergo clonal expansion, and at least 40%, and perhaps all, of the infected cells that persist on cART are the products of clonal expansion [16]. Most of HIV proviruses in patients on long-term cART have significant defects, implying that most infected clones carry defective proviruses. It is possible that clones carrying deleted or hypermutated proviruses are more likely to increase in size than those with proviruses still able to express viral gene products because highly defective proviruses are unlikely to cause cellular toxicity. However, as described by Wiegand et al. [35], not all of the cells in a clonal population express HIV-1 RNA, potentially explaining how some clonal populations persist even if cells that are actively producing virus proteins are eliminated.

To investigate abundance of clones in detail, we developed specific ddPCR approaches to quantify levels of specific HIV clones. For this purpose, we investigated the proliferation of a clone of cells that had a provirus integrated in *HORMAD2*, which accounted for a substantial (c. 25%) of all proviruses in PBMC in participant AVBIO2_21. We found that cells in this clone underwent a rapid expansion over a short period, were maintained for years despite multiple treatment interruptions, and contributed to the shift in the proviral landscape. The *HORMAD2* provirus consists of a solo LTR, which was likely formed by homologous recombination between the identical LTR sequences at each end of a provirus which had two LTRs [77,84,85]. Although not previously described in HIV-infected individuals in vivo, such solo LTRs are well described in other retrovirus contexts [77], including tumors [78], and comprise as much as 90% of the endogenous proviruses in the human genome [86]. The frequency of HIV solo LTRs in patients is not known. Because solo LTRs do not contain any HIV coding sequences, cells containing them cannot produce any HIV proteins, and will not be an immunologic target, likely facilitating their persistence during cART. The majority of the cells in the *HORMAD2* clone were effector memory cells. The forces responsible for the expansion of this clone of cells are not known, although a number of immune mechanisms, including response to antigen stimulation, may contribute. There is selection for cells with proviruses in *BACH2*, *MKL2*, and *STAT5B* [16,31,32,87] and it is likely that the presence of the proviruses disrupt the normal expression of the genes, which plays a role in proliferation and/or survival of the infected cell. By contrast, *HORMAD2* is involved in meiotic crossing over, not lymphocyte regulation, and its expression in lymphocytes has not been reported in the Genotype-Tissue Expression project analysis of human derived tissues [86]. It is possible that the *HORMAD2* provirus represents a marker tagging of cells that clonally expanded for some other reason, such as response to a specific antigen. The large clonal expansion was detected in a single study participant, and it is not known how common expansions of this magnitude are in infected individuals. Fromentin et al. identified a massively expanded cell clone harboring a deleted provirus in an HIV-infected individual after 3 years on cART [88]; additional study of more individuals undergoing long-term cART will be useful to determine the frequency of such events.

Determining the levels of HIV proviruses in individuals undergoing cART is essential for comprehensive analysis of HIV persistence during cART because both replication competent and defective proviruses may contribute to HIV pathogenesis during cART. Replication competent HIV variants comprise a reservoir of HIV that can rebound if cART is interrupted, and some defective viruses can produce portions of HIV proteins that may contribute to the immune activation that persists during long term cART [25,26]. Immune activation is associated with mortality in HIV infected individuals on cART [44,89], and understanding determinants of activation is essential. Strategies, such as ddPCR, that are both sensitive in detecting and precise in the quantification of HIV proviruses have a number of advantages for studies of HIV pathogenesis during cART. Recent studies by Bruner and coworkers demonstrated the utility of ddPCR approaches in quantifying proviruses that are likely to be replication competent [45]. As demonstrated here, multiplexed ddPCR approaches that can simultaneously quantify the total number of HIV proviruses (LTR) and proviruses with that carry large deletions and/or major defects. This approach for characterizing the kinetics of proviral decay during long term cART will be useful in additional persistence studies, including investigating the anatomic distribution of deleted proviruses, as well as evaluating the effectiveness of interventions intended to control or eradicate HIV. The effects of latency reversing agents [90] on HIV-1 DNA copy numbers may be quite different in populations of cells that carry highly deleted proviruses, compared with cells that carry non-deleted proviruses. Thus, analyses of the efficacy of eradication strategies that rely only on bulk proviral DNA to evaluate strategies to reduce or eliminate the intact proviral DNA reservoir, are likely to give misleading, and possibly false-negative, results. In addition, multiplexed analyses will be useful in quantifying HIV proviruses in studies of individuals undergoing treatment interruptions. Hiener and coworkers reported finding significant differences in levels of proviruses in individuals with post treatment control of HIV [83]; analyses of similar cohorts using multiplexed assays will determine whether, and to what degree, there is relative enrichment of deleted proviruses takes place during post-treatment control.

Our study used ddPCR approaches that do not distinguish integrated from unintegrated HIV species. During ongoing viral replication prior to cART, PBMC are likely to contain high levels of unintegrated HIV-1 DNA and, after introduction of cART, levels of unintegrated DNA decline [23]. Early studies using alu PCR techniques to quantify integrated DNA suggested substantial levels of unintegrated DNA were present during cART [91]. More recently Strongin and coworkers [92] and Lada et al. [93] have quantified integrated HIV DNA in individuals undergoing cART using an electrophoretic approach to enrich for integrated HIV DNA; these newer techniques are more sensitive than Alu PCR approaches, and showed that, during cART, the majority of cell associated viral DNA is integrated. Using standard qPCR approaches, Besson and colleagues found that the levels of 2 LTR circular HIV DNA are very low in patients on long term cART relative to pretherapy [23]. Therefore, any bias due to unintegrated forms cannot explain the observed enrichment of deleted proviruses, especially during prolonged therapy.

The timing of the dramatic shift in the proviral landscape that we identified during cART has important implications for understanding HIV pathogenesis. The increase in the fraction of deleted/highly defective proviruses occurs only after 1–2 years of cART, when most of the infected cells have already been eliminated, and when the total level of HIV-1 DNA remains relatively constant. These data suggest that forces driving HIV persistence remain roughly balanced (Figure 7). Cell populations with HIV proviruses persist, clonally expand, and undergo profound shifts in composition during long-term cART; understanding their role in immune responses and activation will provide new insights that will be useful in identifying and evaluating strategies to eliminate or control HIV infection.

## Supplementary Materials

The following are available online at https://www.mdpi.com/1999-4915/12/2/136/s1, Figure S1: All HIV-1 DNA regions decline upon cART initiation in individual participants, Figure S2: Median HIV-1 DNA copies/million PBMC before and after cART initation, Figure S3: HIV-1 LTR and gag DNA decline upon cART initiation, Figure S4: Multiplexed ddPCR assay to simultaneously measure HIV-1 LTR DNA and HIV-1 gag DNA, Figure S4: Multiplexed ddPCR assay to simultaneously measure HIV-1 LTR DNA and HIV-1 gag DNA, Figure S5: The ratio of HIV-1 LTR DNA to internal HIV-1 DNA sequences during cART. HIV viral load (HIV-1 RNA copies per mL plasma) is shown in black, open symbols represent values below the limit of detection and LTR, Figure S6: Characterization strategy and structure of provirus in HORMAD2 gene from expanded cell clones. Primers are depicted as arrows, Figure S7: Gating for CD4 T Naïve, CTM, and EM Cell Subsets. PBMC, Table S1: Pre-ART study participant demographics and clinical information on study entry, Table S2: Median HIV-1 DNA copies per million PBMC for each primer/probe set from all participants at timepoints before and after cART initiation, Table S3: Unique integration sites from HIV-infected cell clones found in CTM and EM subsets of one individual, Table S4: Primer probe sets for DNA quantification by ddPCR, Table S5: *HORMAD2* proviral characterization strategy primer sequences.
