# Supplementary material for: Dynamic Shifts in the HIV Proviral Landscape During Long Term Combination Antiretroviral Therapy: Implications for Persistence and Control of HIV Infections
Source: Viruses. 2020 Jan 25;12(2):136. doi: 10.3390/v12020136 (PMC7077288; doi:10.3390/v12020136)
Supplement: Supplementary file 1 [file viruses-12-00136-s001.pdf]

Supplemental Figure 1

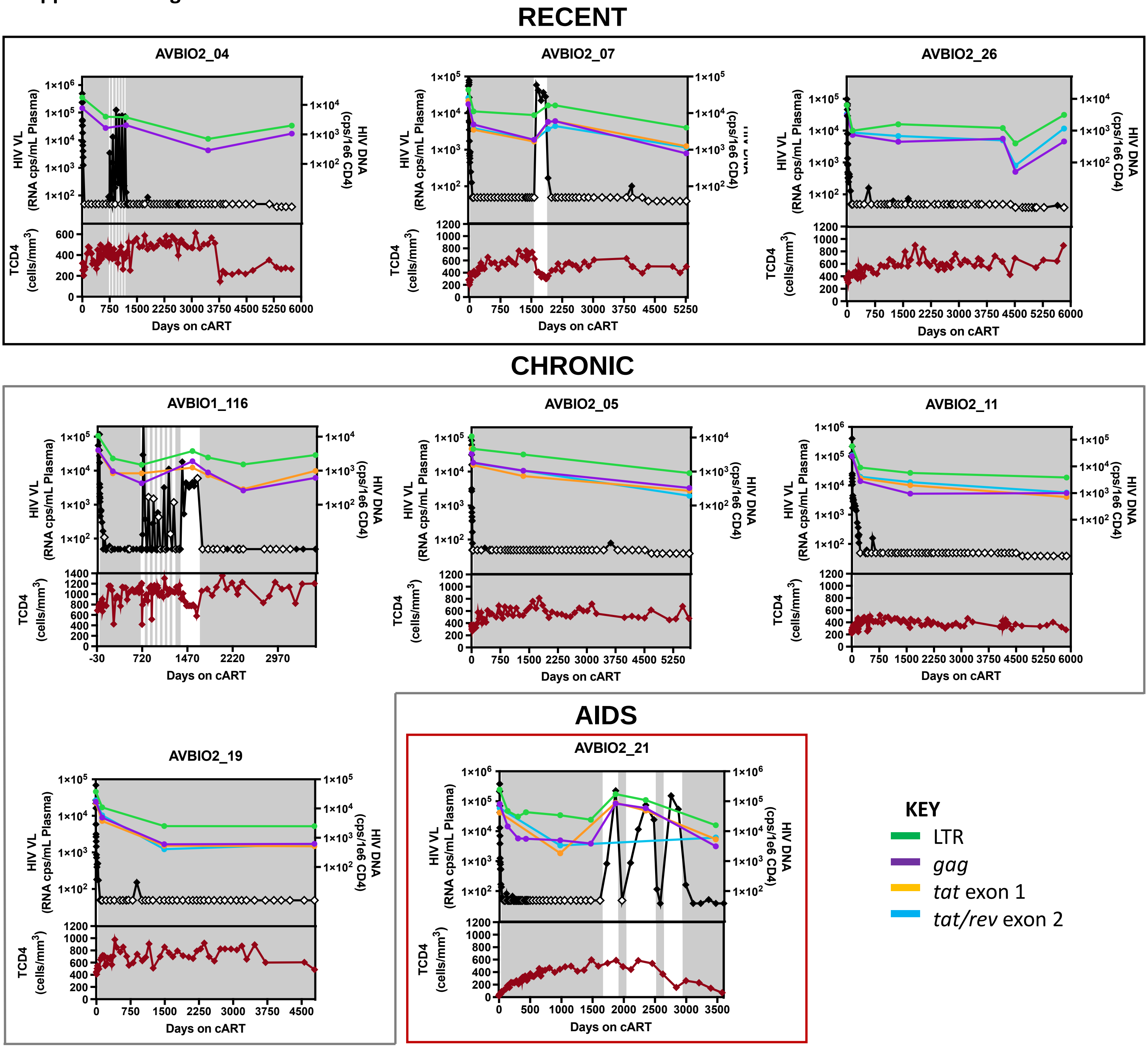

Supplemental Figure 2.

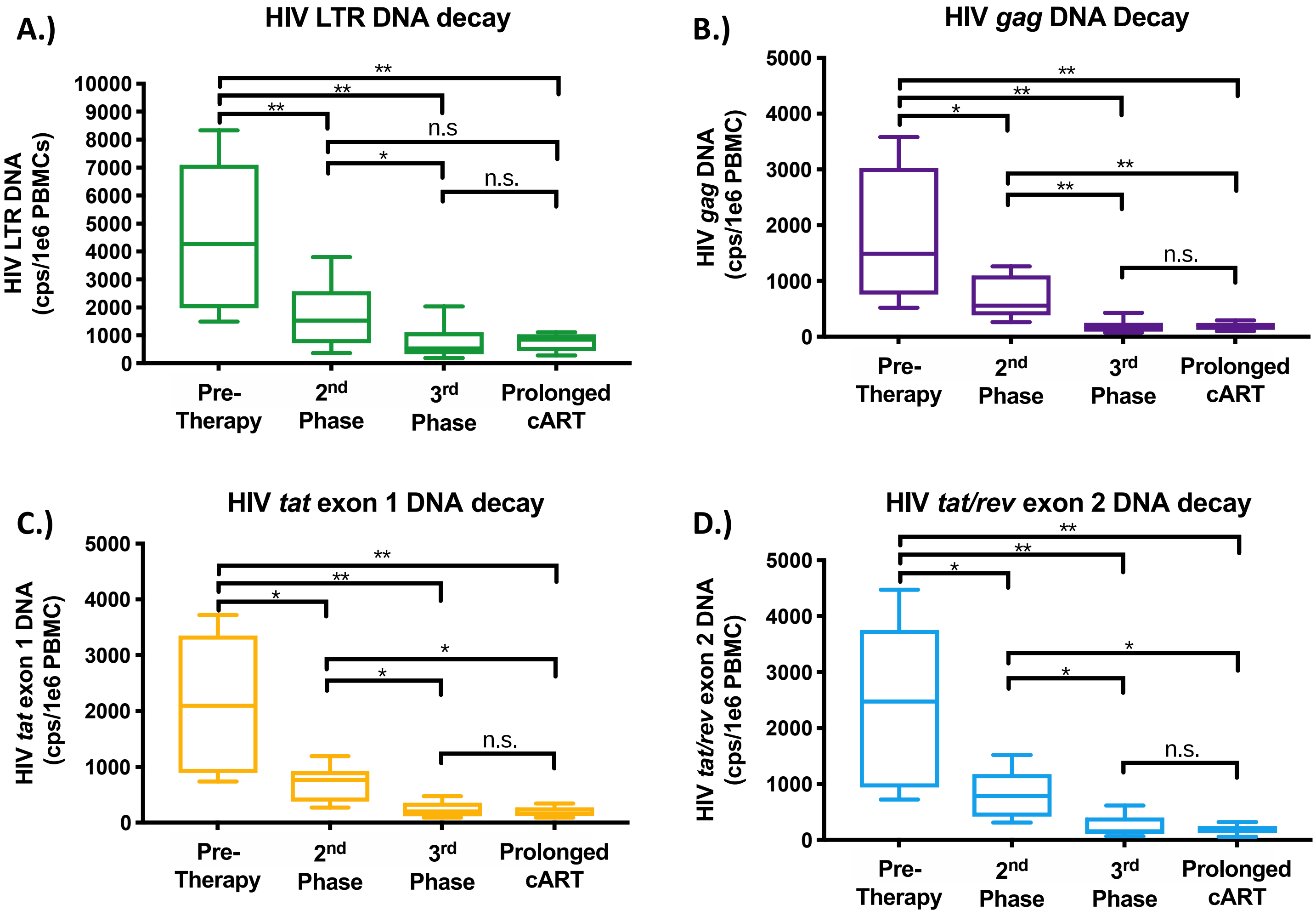

Supplemental Figure 3a

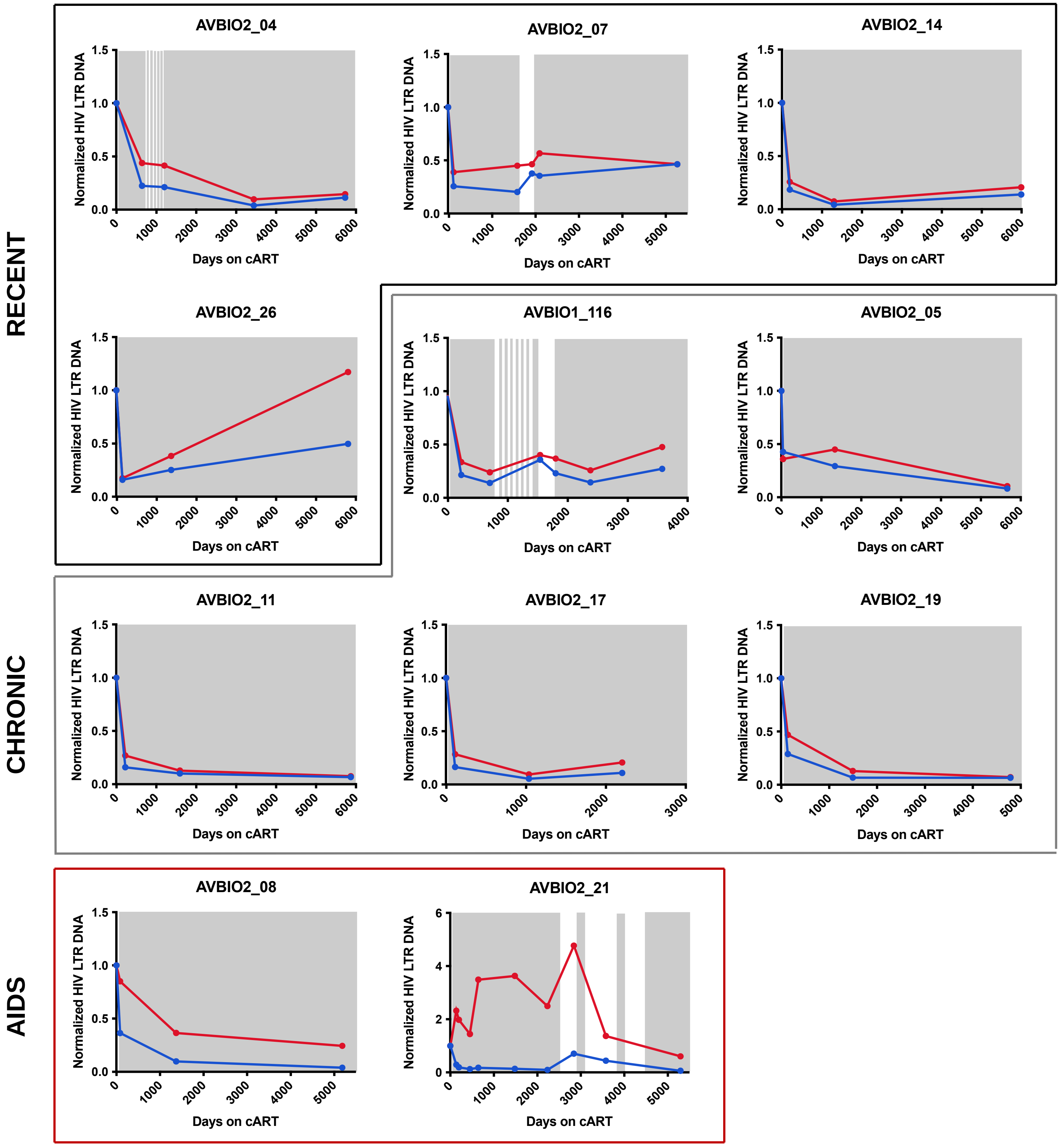

Supplemental Figure 3b

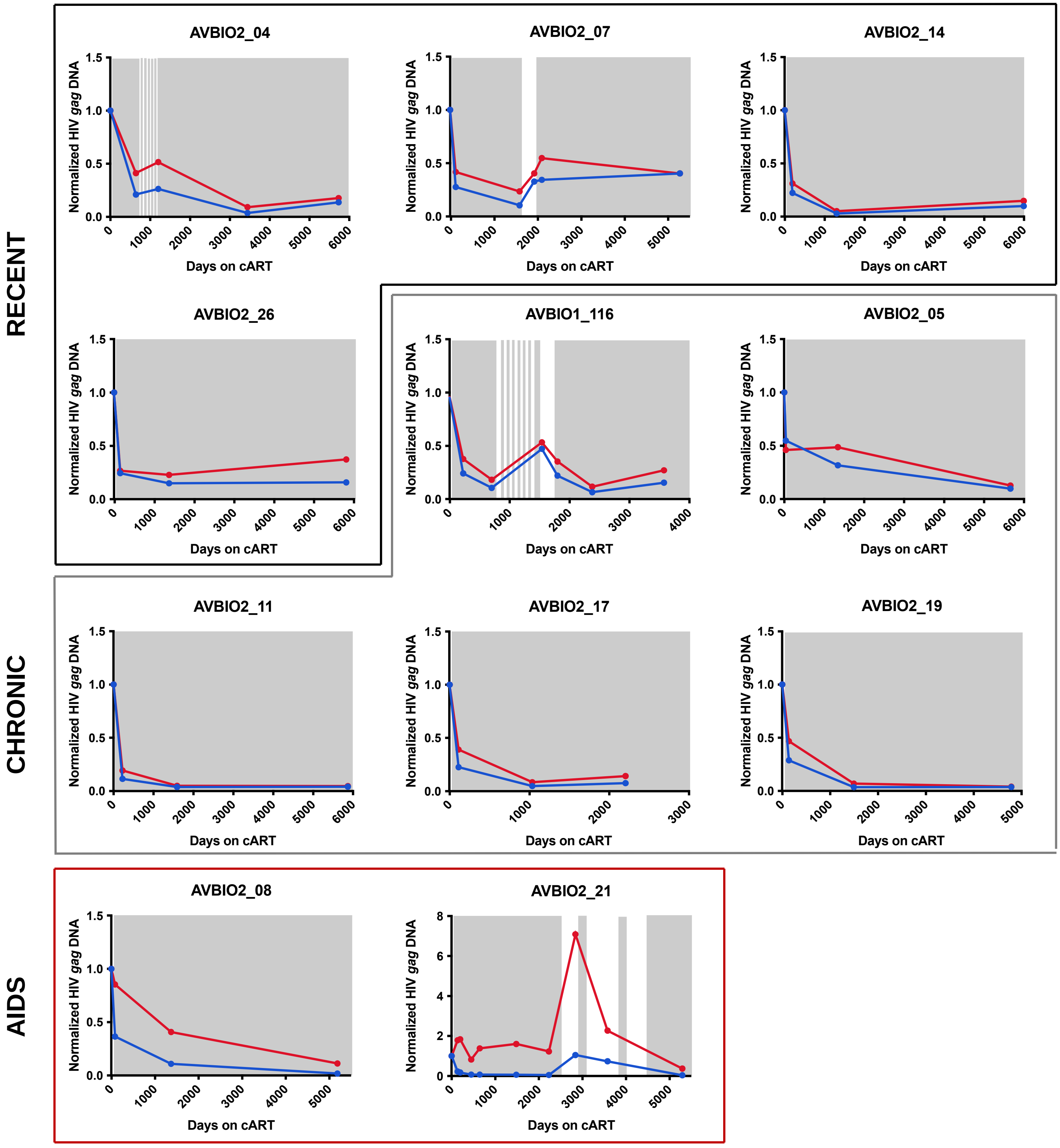

Supplemental Figure 4

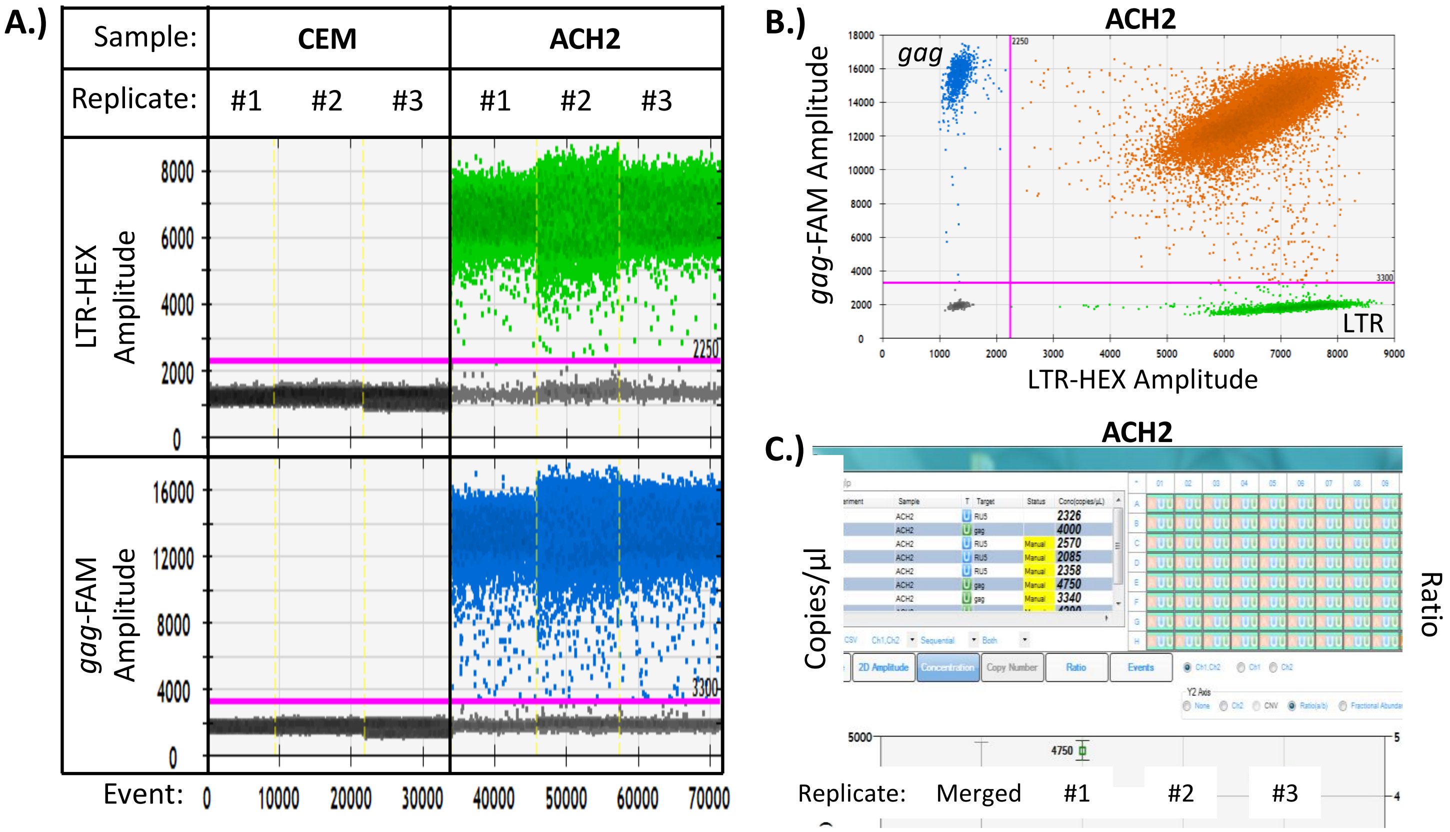

Supplemental Figure 5

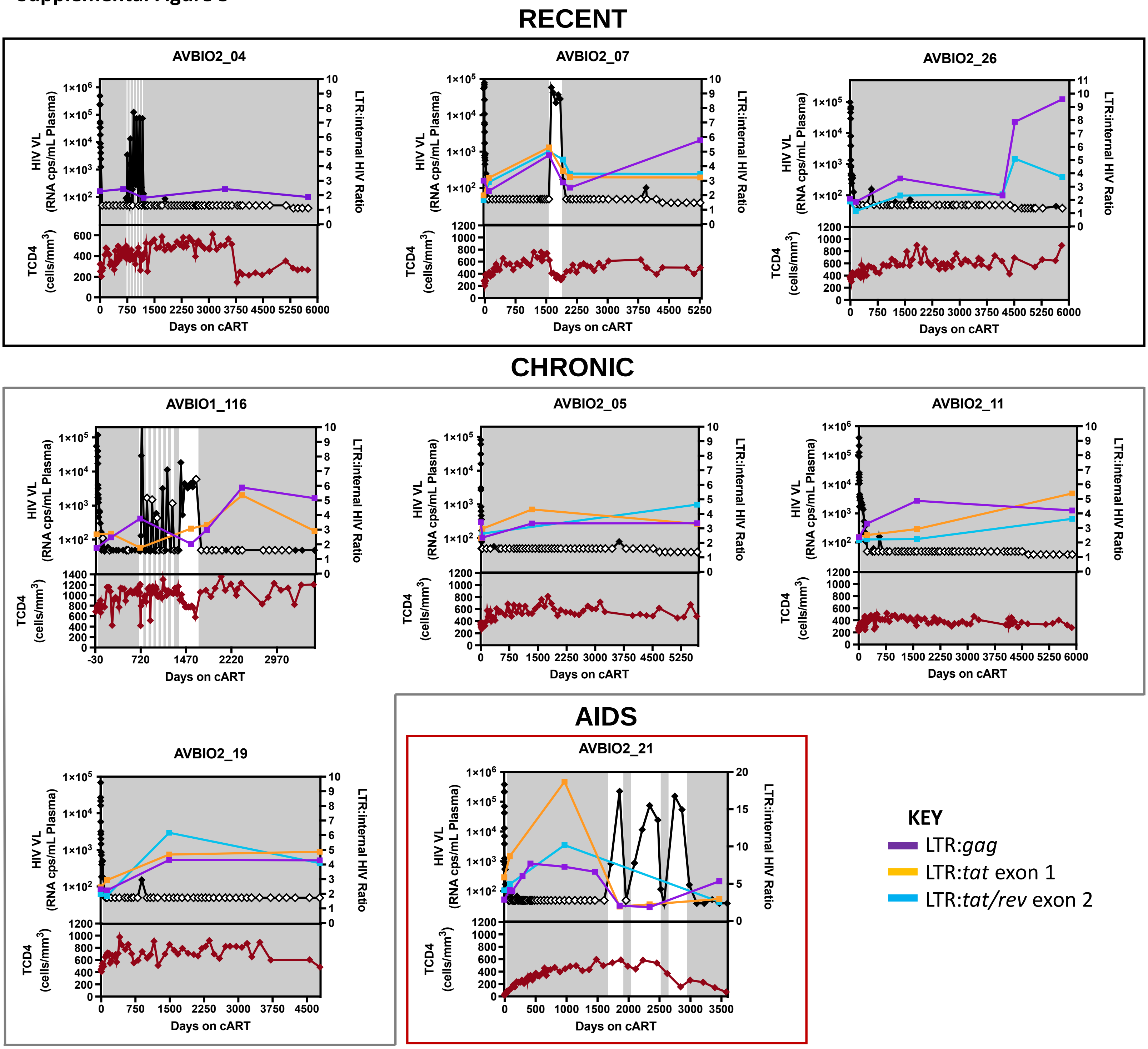

Supplemental Figure 6

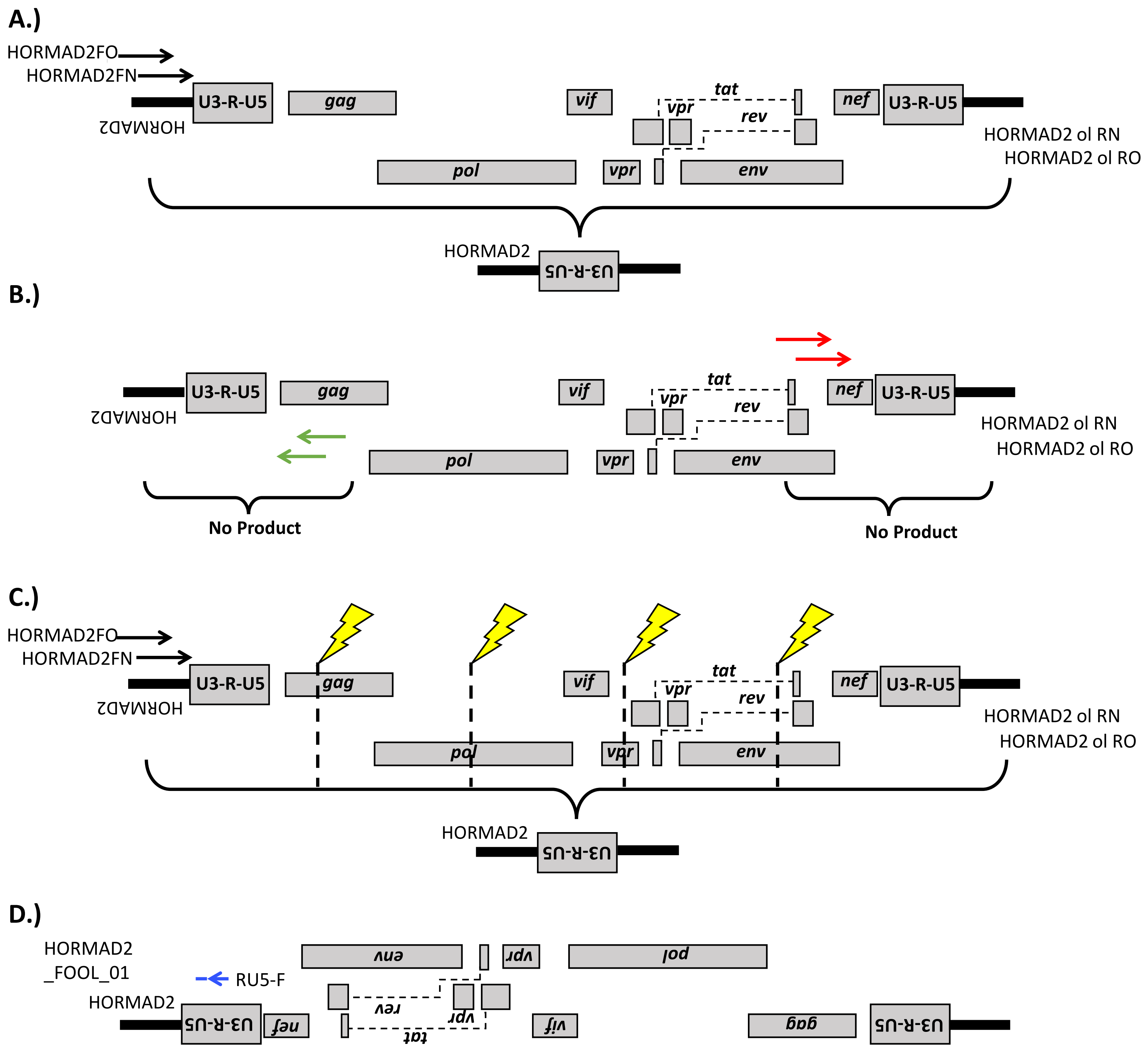

Supplemental Figure 7

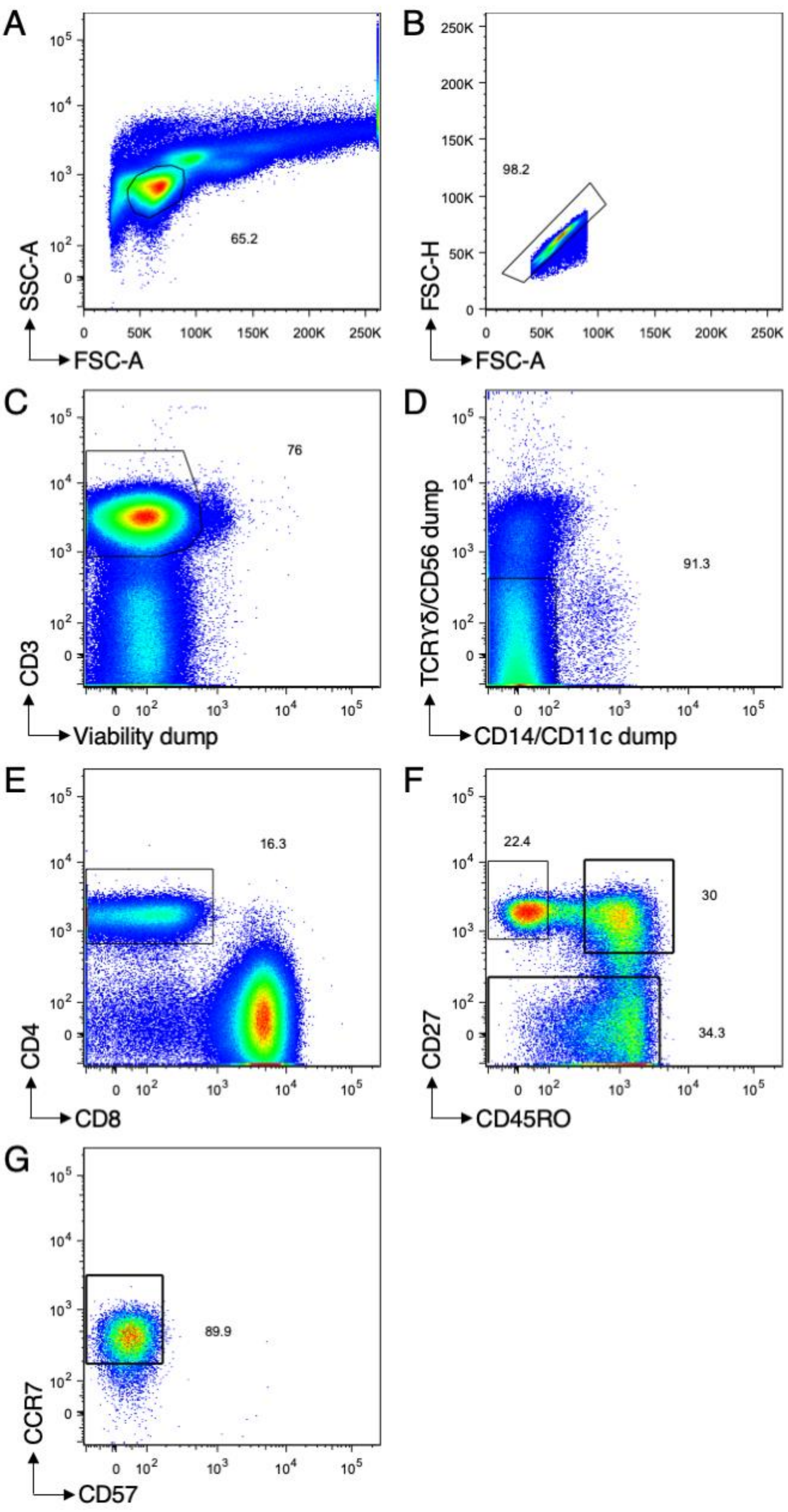

Supplemental Table 1. Pre-cART study participant demographics and clinical information on study entry.

Baseline Infection Status	PID	Age upon cART initiation	Time since diagnosis (years)‡	Gender	Race/Ethnicity	Risk	Baseline HIV RNA (Log10 cps/mL)	Baseline TCD4 (cells/μL)	Baseline CD4%
*Recent HIV infection (<1 year)	AVBIO2_04	39	0.52	M	White/Hispanic	MSM/Shared Needles for Tattoos	5.38	321	28%
	AVBIO2_07	33	0.04	M	White/Non Hispanic	MSM	4.84	282	20%
	AVBIO2_14	32	0.21	M	White/Non Hispanic	MSM	5.86	479	29%
	AVBIO2_26	46	0.27	M	Black/Not Hispanic	MSM	4.78	379	4%
Chronic HIV Infection (>1 year)	AVBIO1_116	37	0.19	M	White/Non Hispanic	MSM	4.74	687	23%
	AVBIO2_05	34	0.29	M	Black/Not Hispanic	Bisexual	4.53	373	20%
	AVBIO2_11	39	0.85	M	White/Non Hispanic	MSM	5.1	283	17%
	aAVBIO2_17	25	1.01	F	Black/Not Hispanic	Heterosexual	4.31	258	16%
	AVBIO2_19	29	0.42	M	Black/Not Hispanic	Heterosexual	4.43	440	25%
HIV/AIDS	AVBIO2_08	50	0.11*	M	Black/Not Hispanic	Bisexual	4.88	62	6%
	bAVBIO2_21	51	14.8	M	Black/Not Hispanic	MSM	5.58	23	2%
MEDIAN:		37	0.36				4.84	321	20%

‡Years between the date of first positive Western Blot and cART intiation
*History of recent HIV infection based on prior non-reactive ELISA or documented seroconversion event
aPatient 2 in Maldarelli et al. Science 2014 [15]
bPatient 3 in Maldarelli et al. Science
2014 [15]

Supplemental Table 2. Median HIV-1 DNA copies per million PBMC for each primer/probe target from participants at timepoints before and after cART initiation.

	Timepoint			
	Pre-cART	Suppression	3rd Phase	Long-term
Days on continuous suppressive cART				
Median (Q1-Q3)	NA	139 (110-181)	1342 (868-1433)	5423 (4148-5825)
Min-Max	NA	40-224	642-1590	2205-5990
Total CD4 count (cells/mm3)				
Median (Q1-Q3)	321 (252-410)	421 (339-615)	573 (425-728)	486 (455-616)
Min-Max	23-687	145-1074	230-1173	278-895
HIV LTR DNA (copies/1e6 PBMCs)				
Median (Q1-Q3)	3659 (2164-6690)	1887 (854-2749)	812 (425-1311)	843 (649-1042)
Min-Max	1498-8332	361-3803	231-5206	290-4504
HIV gag DNA (copies/1e6 PBMCs)				
Median (Q1-Q3)	1199 (910-2647)	711 (422-1036)	194 (121-390)	229 (122-257)
Min-Max	715-3579	265-1263	93-675	102-726
HIV tat exon 1 DNA (copies/1e6 PBMCs)				
Median (Q1-Q3)	1917 (769-2870)	747 (402-783)	197 (105-273)	172 (120-169)
Min-Max	625-3719	270-1188	70-471	88-345
HIV tat/rev exon 2 DNA (copies/1e6 PBMCs)				
Median (Q1-Q3)	2463 (889-3541)	710 (455-885)	157 (126-255)	189 (139-220)
Min-Max	795-4475	312-1517	71-613	62-321

Supplemental Table 3. Unique integration sites from HIV-infected cell clones found in CTM and EM subsets of one individual.

	CTM			EM			
Gene	Number	Frequency	Copies/ml	Number	Frequency	Copies/ml	Fisher's Exact p-value
ANKRD13D	4	1.2%	26.9	0	0.0%	<12	0.51
KCTD13	3	0.9%	20.1	0	0.0%	<12	0.18
ZNF564	1	0.3%	6.6	1	0.3%	11.2	1
FCGBP	1	0.3%	6.6	4	1.1%	44.3	0.38
HORMAD2	1	0.3%	6.6	65	18.1%	720.1	0.0001***
TSG101	0	0.0%	<6.9	18	5.0%	199.4	0.0001***
TGFBR3	0	0.0%	<6.9	12	3.3%	132.8	0.0005***
YPEL1	0	0.0%	<6.9	10	2.8%	110.8	0.0020**
SLX4	0	0.0%	<6.9	9	2.5%	99.7	0.0040**
CKAP5	0	0.0%	<6.9	9	2.5%	99.7	0.0040**
HELB	0	0.0%	<6.9	6	1.7%	66.6	0.0080*
DNAH6	0	0.0%	<6.9	5	1.4%	55.4	0.06
C7orf59a	0	0.0%	<6.9	4	1.1%	44.3	0.13
GRIP1	0	0.0%	<6.9	4	1.1%	44.3	0.13
PPIL2	0	0.0%	<6.9	4	1.1%	44.3	0.13
PELP1	0	0.0%	<6.9	4	1.1%	44.3	0.13
KCTD13	0	0.0%	<6.9	3	0.8%	33.1	0.25
EMR4P	0	0.0%	<6.9	3	0.8%	33.1	0.25
GNB1	0	0.0%	<6.9	3	0.8%	33.1	0.25
CPEB2	0	0.0%	<6.9	3	0.8%	33.1	0.25
PRKCB	0	0.0%	<6.9	3	0.8%	33.1	0.25
ANKRD13D	0	0.0%	<6.9	2	0.6%	22.3	0.5
C7orf59b	0	0.0%	<6.9	2	0.6%	22.3	0.5
GSTO1	0	0.0%	<6.9	2	0.6%	22.3	0.5
MIR1208	0	0.0%	<6.9	2	0.6%	22.3	0.5
SMARCE1	0	0.0%	<6.9	2	0.6%	22.3	0.5
TGFBR3	0	0.0%	<6.9	2	0.6%	22.3	0.5

Supplemental Table 4. Primer probe sets for DNA quantification by ddPCR.

Target	Primer Name	Sequence	Reference
CCR5	hCCR5-F1	5'-CCA GAA GAG CTG AGA CAT CCG-3'	Thomas, J.A. et al., 2006, Virology
	hCCR5-R1	5'-GCC AAG CAG CTG AGA GGT TAC T-3'	
	FAM-hCCR5-P01	5'-/56-FAM/-TCC CCT ACA AGA AAC TCT CCC CGG-/3BHQ_1/-3'	
LTR	RU5-F	**5'-CTT AAG CCT CAA TAA AGC TTG CC-3'**	Anderson, E.M. et al., 2018 Curr Protoc Microbiol
	RU5-R	**5'-GGA TCT CTA GTT ACC AGA GTC-3'**	
	RU5-Probe	**5'-/5HEX/ -AGT AGT GTG TGC CCG TCT G-/ZEN//3IaBkFQ/-3'**	
gag	HIV SCA 6F	**5'-CAT GTT TTC AGC ATT ATC AGA AGG A-3'**	Palmer, S. et al., 2003, J. Clin Microbiol
	HIV SCA 84R	**5'-TGC TTG ATG TCC CCC CAC T-3'**	
	HIV SCA probe 32t ZEN	**5'-/56-FAM/-CCA CCC CAC AAG ATT TAA ACA CCA TGC TAA-/ZEN//3IaBkFQ/-3'**	
tat Exon 1	TatRev1_F	**5'-GCA TCC AGG AAG TCA GCC TA-3'**	Anderson, E.M. et al., 2018 Curr Protoc Microbiol
	TatRev1_R	**5'-CTT CGT CGC TGT CTC CGC-3'**	
	TatRev1_probe	**5'-/5HEX/ -TC TTC CTG CCA TAG GAG ATG C-/3BHQ_1/-3'**	
tat/rev Exon 2	TatRev2_F	**5'- GTG AAT AGA GTT AGG CAG GGA TA-3'**	Anderson, E.M. et al., 2018 Curr Protoc Microbiol
	msRNA-R	**5'-GTC TCT GTC TCT CTC TCC ACC-3'**	
	msRNA Probe	**5'-/56-FAM/-TCG GGC CTG TCG GGT CC-/3BHQ_1/-3'**	
HORMAD2	RU5-F	**5'-CTT AAG CCT CAA TAA AGC TTG CC-3'**	
	HORMAD2_FOOL_01	5'-CCT GTG CCT AGG GTA GAT AAT AC**T GCT AGA G**-3'	
	RU5-Probe	**5'-/5HEX/ -AGT AGT GTG TGC CCG TCT G-/ZEN//3IaBkFQ/-3'**	
AMBI1	RU5-F	**5'-CTT AAG CCT CAA TAA AGC TTG CC-3'**	
	AMBI1_ROOL_01	5’-CGC TTC ATT AGC ACT TAC **TGC TAG AG-3’**	
	RU5-Probe	**5'-/5HEX/ -AGT AGT GTG TGC CCG TCT G-/ZEN//3IaBkFQ/-3'**	

Bold Text indicates HIV specific sequences

Supplemental Table 5. *HORMAD2* proviral characterization strategy primers. Nested primer sets for the characterization of a unique provirus integrated into the *HORMAD2* gene of participant AVBIO2_21. Bold text indicates HIV specific sequence.

HORMAD2 Integrant Mapping Strategy

Strategy	Primer Name	Sequence
Forward Human Outer	HORMAD2FO	CTCAACTGCTATGCCTAGATAGCG
Forward Human Nested	HORMAD2FN	GATATAGCCAGCCTGTGCC
Reverse Overlapping Outer	HORMAD2 ol RO	CAAATAGTCCAAGGGTATT**TGGAAGGG**
Reverse Overlapping Nested	HORMAD2 ol RN	GGTATT**TGGAAGGGCTAATTTACTCCCA**
